# Supplementary material for: IL-33-ST2 axis regulates myeloid cell differentiation and activation enabling effective club cell regeneration
Source: Nat Commun. 2020 Sep 22;11:4786. doi: 10.1038/s41467-020-18466-w (PMC7508874; doi:10.1038/s41467-020-18466-w)
Supplement: Supplementary file 1 — Supplementary Information [file 41467_2020_18466_MOESM1_ESM.pdf]

IL-33-ST2 axis regulates myeloid cell differentiation and activation enabling effective club cell regeneration

Dagher R, Copenhaver AM, Besnard V, Berlin A, Hamidi F, Maret M, Wang J, Qu X, Shrestha Y, Wu J, Gautier G, Raja R, Aubier M, Kolbeck R, Humbles AA and Pretolani M

Supplementary information

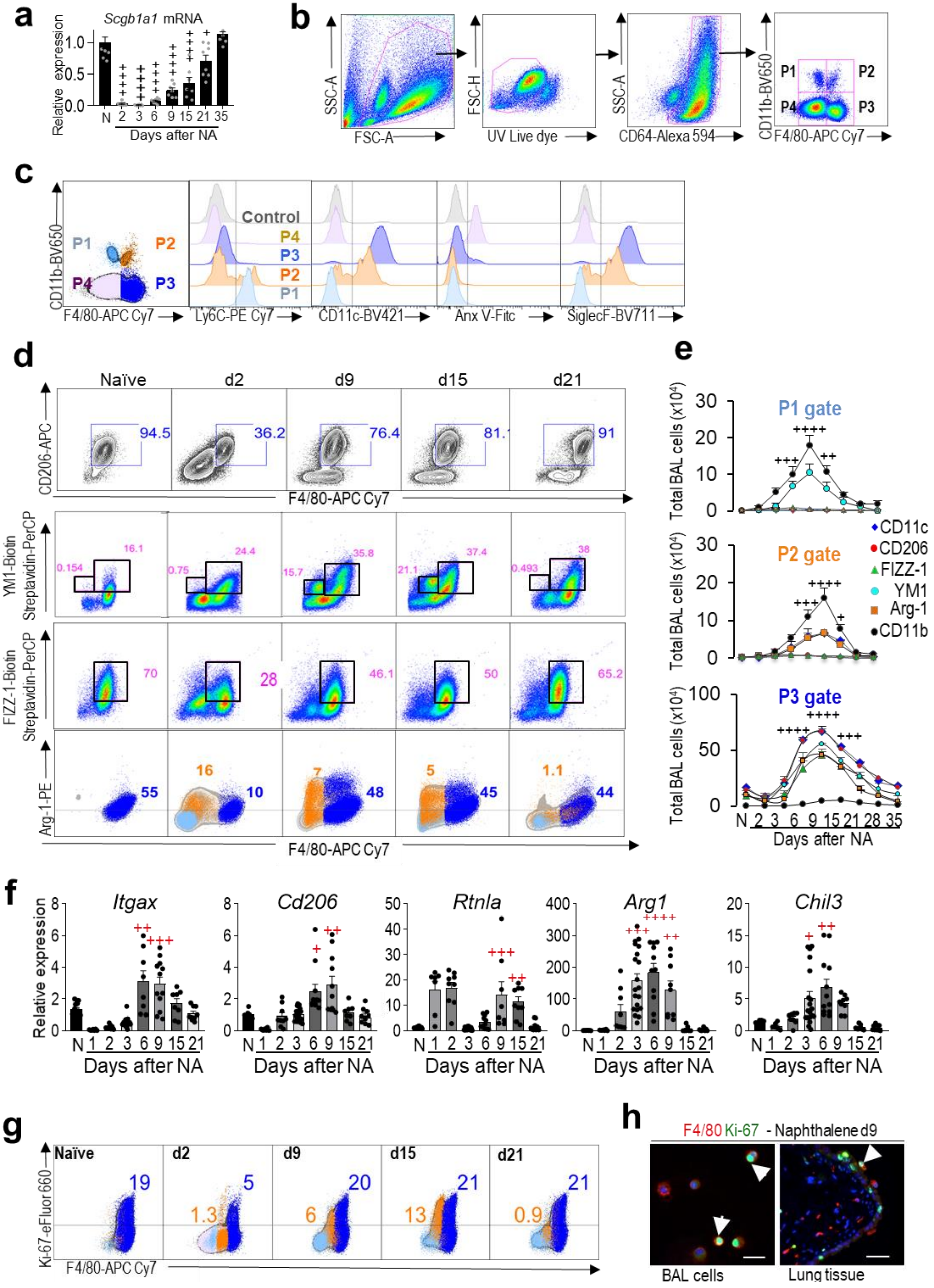

a
Scgb1a1 mRNA
Relative expression
Days after NA
b
SSC-A
FSC-A
FSC-H
UV Live dye
CD64-Alexa 594
CD11b-BV650
F4/80-APC Cy7
P1
P2
P3
P4
c
Control
Ly6C-PE Cy7
CD11c-BV421
Anx V-Fitc
SiglecF-BV711
d
Naïve
d2
d9
d15
d21
CD206-APC
YM1-Biotin Streptavidin-PerCP
FIZZ-1-Biotin Streptavidin-PerCP
Arg-1-PE
e
P1 gate
P2 gate
P3 gate
Total BAL cells (x10⁴)
CD11c
CD206
FIZZ-1
YM1
Arg-1
CD11b
f
Itgax
Cd206
Rtnla
Arg1
Chil3
g
Ki-67-eFluor 660
h
F4/80 Ki-67 - Naphthalene d9
BAL cells
Lung tissue

Supplementary Figure 1. Myeloid cell phenotyping in BAL and lung in the airways of NA-injured mice.

WT mice were either naïve (N) or treated with NA. Lung cells and BAL cells were analyzed at various days after NA administration. **(a)** Levels of *Scgb1a1* mRNA in total lung homogenates from NA-treated WT mice euthanized at the indicated time points. **(b-c)** Gating strategy for determining and quantifying the phenotype of monocyte/macrophage and subsets from total bronchoalveolar lavage (BAL) cells in Figures 1 (g-i), 2d, 4b, 5c and 9g. **(c)** Representative expression of Ly6C, CD11c, SiglecF and Annexin V (Anx V) on the BAL P1-P4 subsets as determined by flow cytometry. **(d)** Representative flow cytometric plots of CD206, F4/80, YM1, FIZZ-1, and Arg-1 expression on BAL cells. Numbers next to flow gates denote percentage of cells positive for the indicated markers.**(e)** Quantification of total numbers of P1, P2, or P3 BAL populations positive for CD11c, CD206, FIZZ-1, YM1, Arg-1, or CD11b. Data are represented as percentage. **(f)** Quantification of mRNA expression from BAL cells for the indicated transcripts. Flow plots are representative. All graphs show individual mice as dots or squares with lines at the mean. **(g)** F4/80 and Ki-67 staining on P1-P3 BAL populations. **(h)** F4/80 (red), Ki-67 (green), and DAPI (blue) immunofluorescent staining of cytospins (BAL cells, left panel) or lung tissue sections (right panel) on day 9 of naphthalene treatment. Bar graphs from n=8 (a), 5 (e) and 8 (f) mice , show mean ± SEM pooled from three independent experiments. $^{+}P < 0.05$, $^{++}P < 0.01$, $^{+++}P < 0.001$ and $^{++++}P < 0.0001$ between NA-treated and naïve WT mice using One-Way ANOVA, Bonferroni post-test. Scale bar in h = 20 μm.

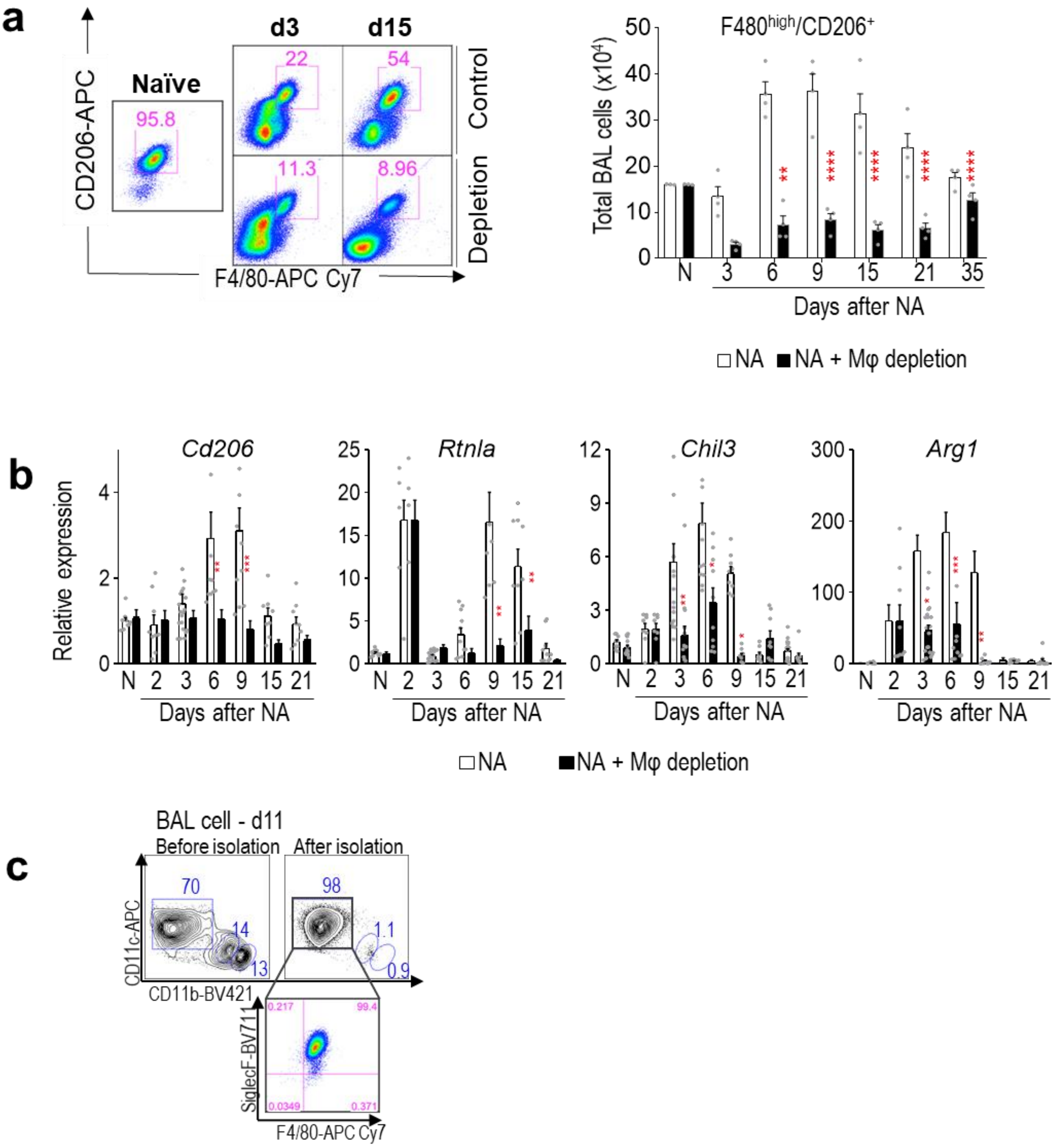
a
CD206-APC
Naïve
95.8
d3
d15
22
54
11.3
8.96
Control
Depletion
F4/80-APC Cy7
F480^high/CD206^+
Total BAL cells (x10^4)
50
40
30
20
10
0
N
3
6
9
15
21
35
Days after NA
NA
NA + Mφ depletion
b
Relative expression
Cd206
Rtnla
Chil3
Arg1
N 2 3 6 9 15 21
Days after NA
NA
NA + Mφ depletion
c
BAL cell - d11
Before isolation
After isolation
70
14
13
98
1.1
0.9
CD11c-APC
CD11b-BV421
SiglecF-BV711
F4/80-APC Cy7
0.217
99.4
0.0349
0.371

Supplementary Figure 2. Airway macrophage depletion in NA-injured mice.

(a) Representative flow cytometric plots showing the frequency of $CD206^{+}$ $F4/80^{+}$ cells in naïve (N), NA-treated WT mice (Control) or NA-injected mice treated with clodronate liposomes (depleted). Numbers denote percentage of cells in gates. In the right panel, quantification of total numbers of $CD206^{+}$ $F4/80^{+}$ cells as gated in the representative FACS plots. **(b)** Quantification of mRNA for the indicated transcripts. **(c)** Representative flow plots showing the frequency of cells used for adoptive transfer as percentage of P3 AAMs before isolation and after isolation. Bar graphs from n=4 (a) and 8 (b) mice, show mean ± SEM pooled from three independent experiments. *$P < 0.05$, **$P < 0.01$, ***$P < 0.001$ and ****$P < 0.0001$ between macrophage-depleted and NA-treated WT mice using Two-Way ANOVA, Bonferroni post-test.

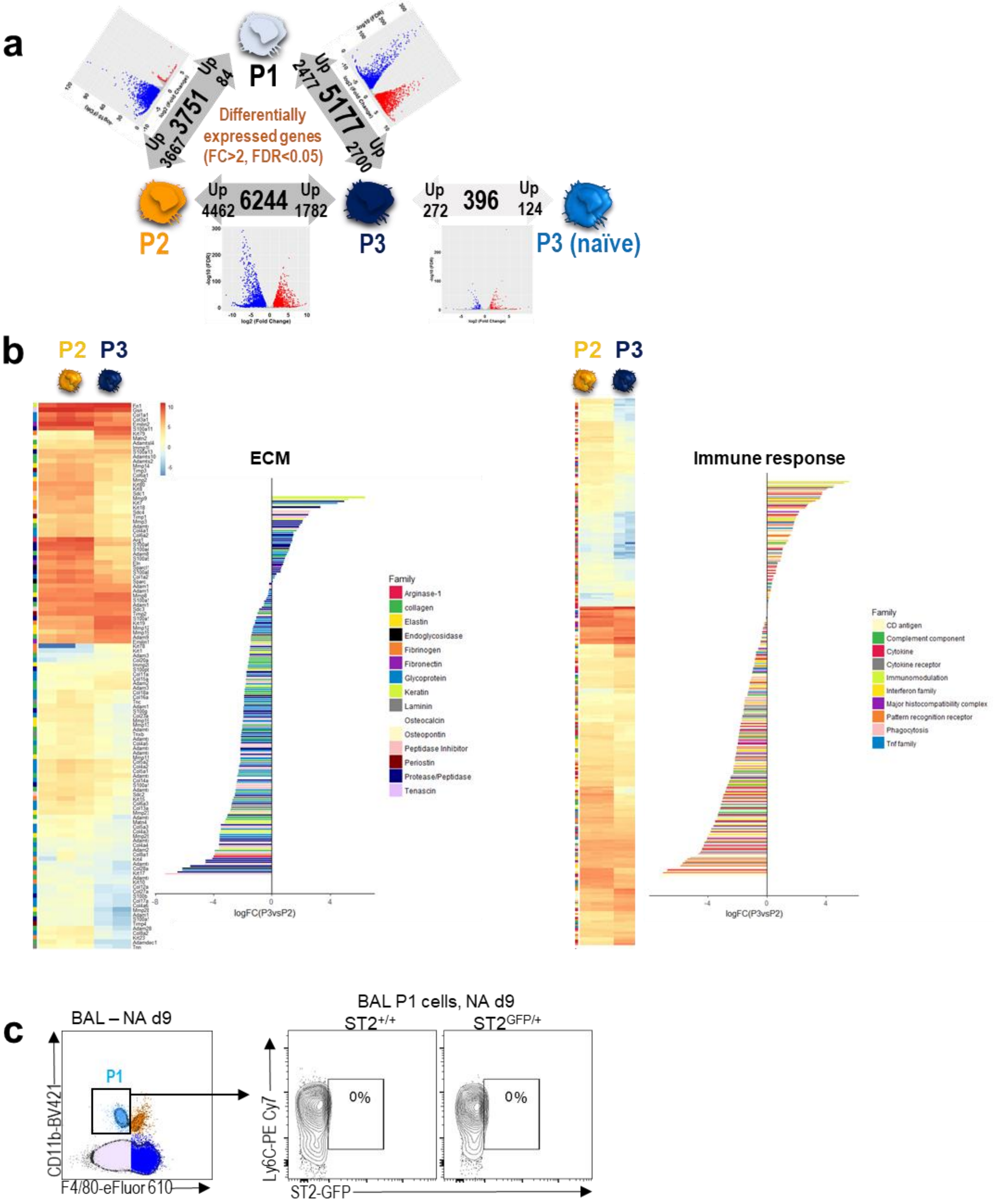
a
P1
Up 84
3751
Up 3667
Up 2477
5177
Up 2700
Differentially expressed genes (FC>2, FDR<0.05)
P2
Up 4462
6244
Up 1782
P3
Up 272
396
Up 124
P3 (naïve)
b
P2 P3
ECM
Family
Arginase-1
collagen
Elastin
Endoglycosidase
Fibrinogen
Fibronectin
Glycoprotein
Keratin
Laminin
Osteocalcin
Osteopontin
Peptidase Inhibitor
Periostin
Protease/Peptidase
Tenascin
logFC(P3vsP2)
Immune response
Family
CD antigen
Complement component
Cytokine
Cytokine receptor
Immunomodulation
Interferon family
Major histocompatibility complex
Pattern recognition receptor
Phagocytosis
Tnf family
logFC(P3vsP2)
c
BAL – NA d9
P1
CD11b-BV421
F4/80-eFluor 610
BAL P1 cells, NA d9
ST2+/+
ST2GFP/+
0%
0%
Ly6C-PE Cy7
ST2-GFP

Supplementary Figure 3. Transcriptomic analysis of lung myeloid cell subsets in the airways of NA-injured mice.

(a) Summary of differentially expressed genes ($P < 0.01$; FC >2) in each pairwise comparison showing differentially expressed genes in red and blue in a volcano plot, the total number of differentially expressed genes in the middle of the bidirectional arrows. **(b)** Heatmaps showing the relative expression of selected genes in P2 and P3 cell subsets that are associated with macrophage-mediated modulation of the epithelial cell niche. These functions include extracellular matrix (ECM) remodeling (left panel) and immune responses (right panel) between P2 and P3 cells. **(c)** FACS quantification as percentage of ST2 expression on P1 cell subsets in BAL. Using GFP ST2 reporter mice, results confirmed no expression of ST2 at the protein level on P1 cells.

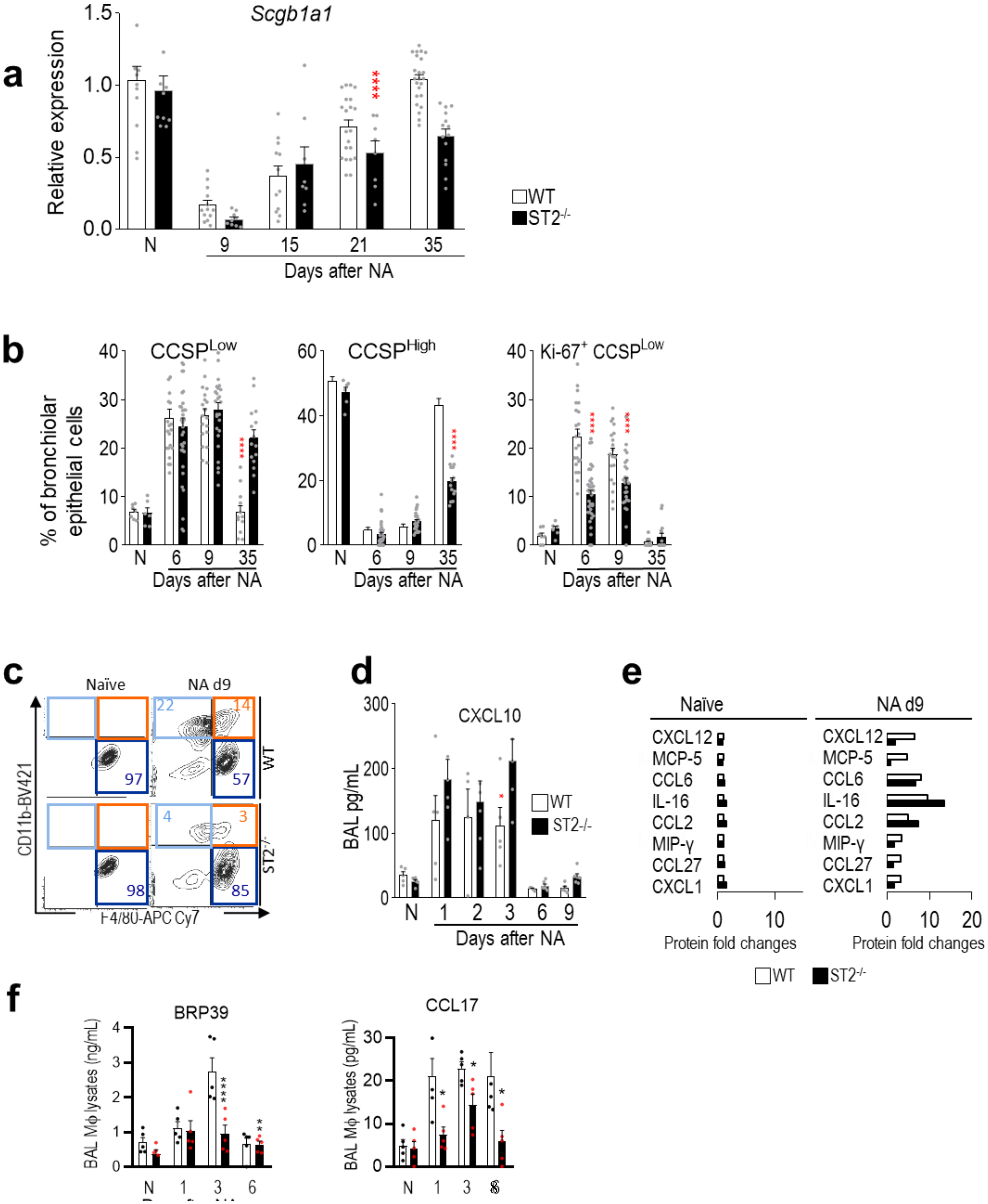
a
Scgb1a1
Relative expression
1.5
1.0
0.5
0.0
N
9
15
21
35
Days after NA
****
WT
ST2-/-
b
% of bronchiolar epithelial cells
CCSP Low
CCSP High
Ki-67+ CCSP Low
Days after NA
c
Naïve
NA d9
WT
ST2-/-
CD11b-BV421
F4/80-APC Cy7
22
14
97
57
4
3
98
85
d
CXCL10
BAL pg/mL
Days after NA
WT
ST2-/-
e
Naïve
NA d9
CXCL12
MCP-5
CCL6
IL-16
CCL2
MIP-γ
CCL27
CXCL1
Protein fold changes
WT
ST2-/-
f
BRP39
BAL Mϕ lysates (ng/mL)
CCL17
BAL Mϕ lysates (pg/mL)

Supplementary Figure 4. ST2 deficient mice exhibit impaired club cell proliferation associated with an alteration in myeloid cell function after club cell injury.

(a) Quantification of *Scgb1a1* mRNA levels in lung homogenates of WT or $ST2^{-/-}$ mice from naïve (N) or treated with naphthalene (NA) and analyzed at the indicated time-points. **(b)** Proportion of club cells expressing CCSP at low and high levels within the bronchioles at indicated time-points (left panels). Right panel, quantification of club cell proliferation, as assessed within the CCSP low population by immunofluorescence staining of Ki-67 in lung tissue sections. **(c)** Representative flow cytometric plots of P1-P3 subsets in BAL cells of WT or $ST2^{-/-}$ mice from naïve or 9 days after treatment with naphthalene (NA d9). Data are represented as percentage. **(d)** Levels of CXCL10 in BAL supernatants. **(e)** Quantification of relative levels of the indicated mediators in BAL macrophage lysates. **(f)** Quantification of BRP-39 and CCL17 in BAL macrophage lysates. Graphs from n=8 (a,b), 6 (d) and 5 (f) mice, shown are mean ± SEM pooled from three independent experiments. * $P < 0.05$, **$P < 0.01$, ***$P < 0.001$ and ****$P < 0.0001$ between $ST2^{-/-}$ and NA-treated WT mice using One-Way (a,b and f) and Two-Way ANOVA (d and e), Bonferroni post-test.

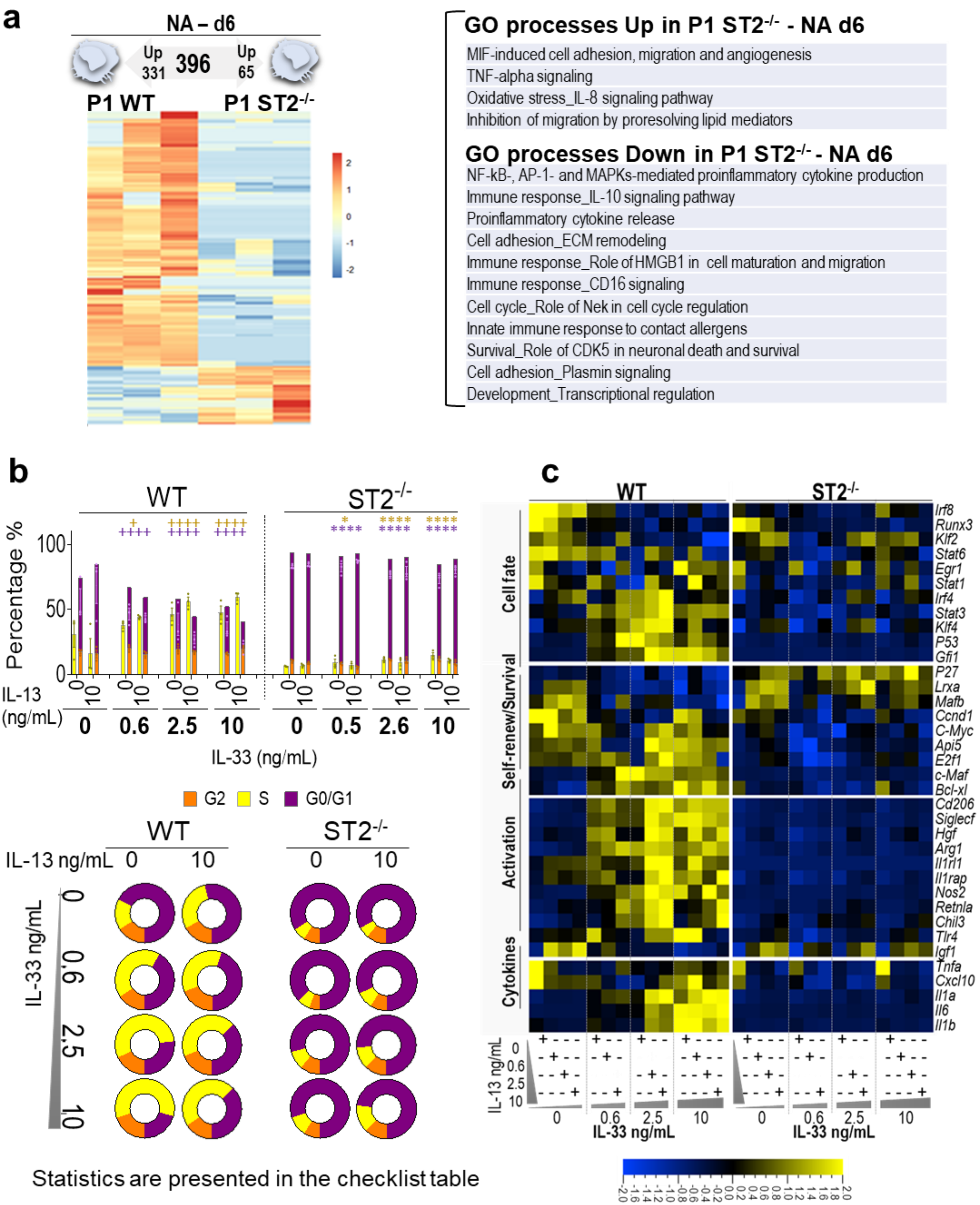
a
NA – d6
Up 331
396
Up 65
P1 WT
P1 ST2-/-
GO processes Up in P1 ST2-/- - NA d6
MIF-induced cell adhesion, migration and angiogenesis
TNF-alpha signaling
Oxidative stress_IL-8 signaling pathway
Inhibition of migration by proresolving lipid mediators
GO processes Down in P1 ST2-/- - NA d6
NF-kB-, AP-1- and MAPKs-mediated proinflammatory cytokine production
Immune response_IL-10 signaling pathway
Proinflammatory cytokine release
Cell adhesion_ECM remodeling
Immune response_Role of HMGB1 in cell maturation and migration
Immune response_CD16 signaling
Cell cycle_Role of Nek in cell cycle regulation
Innate immune response to contact allergens
Survival_Role of CDK5 in neuronal death and survival
Cell adhesion_Plasmin signaling
Development_Transcriptional regulation
b
WT
ST2-/-
Percentage %
IL-13 (ng/mL)
IL-33 (ng/mL)
G2
S
G0/G1
IL-13 ng/mL
IL-33 ng/mL
c
WT
ST2-/-
Cell fate
Self-renew/Survival
Activation
Cytokines
Irf8
Runx3
Klf2
Stat6
Egr1
Stat1
Irf4
Stat3
Klf4
P53
Gfi1
P27
Lrxa
Mafb
Ccnd1
C-Myc
Api5
E2f1
c-Maf
Bcl-xl
Cd206
Siglecf
Hgf
Arg1
Il1rl1
Il1rap
Nos2
Retnla
Chil3
Tlr4
Igf1
Tnfa
Cxcl10
Il1a
Il6
Il1b
IL-13 ng/mL
IL-33 ng/mL
Statistics are presented in the checklist table

Supplementary Figure 5. Transcriptomic profiling of P1 cells in ST2 deficient mice after NA injury; ST2 deficiency alters cell cycle and self-renewal of bone marrow-derived macrophages and also alters the phenotype of bone marrow-derived macrophages.

(a) Heatmap of differentially expressed genes ($P < 0.01$; FC >2) in P1 showing differentially expressed genes (6 days post-NA) in WT and $ST2^{-/-}$ on the left and right of the bidirectional arrow. The total number of differentially expressed genes (396) is presented in the middle of the bidirectional arrow. The GO pathway analysis associated with the genes differentially expressed in WT versus $ST2^{-/-}$ are illustrated on the right side of the heatmap. Scale bar on the right denotes relative log_2 differences in gene expression for each row. **(b)** BMDMs were cultured alone or M-CSF with the indicated concentrations of IL-33 and/or IL-13 for 3 days. Quantification of G0/G1, S, and G2 phases in WT and $ST2^{-/-}$ BMDMs as determined by gating in Fig. 7a. Graphs show mean ± SEM from BMDMs separately cultured from three individual mice. *$P < 0.05$, **$P < 0.01$, ***$P < 0.001$ and ****$P < 0.0001$ between IL13 or/and IL-33-stimulated WT and $ST2^{-/-}$ BMDMs using Two-Way ANOVA (b), Bonferroni post-test. **(c)** Heat-map constructed from Fluidigm analysis of mRNA transcripts for the indicated genes from cultured WT and $ST2^{-/-}$ BMDMs. Scale bar to the left denotes relative log_2 differences in gene expression for each row. Disc graphs denote mean only. Bar graphs in b show means ± SEM. Samples are bone marrow treated and cultured separately from three individual mice per genotype.

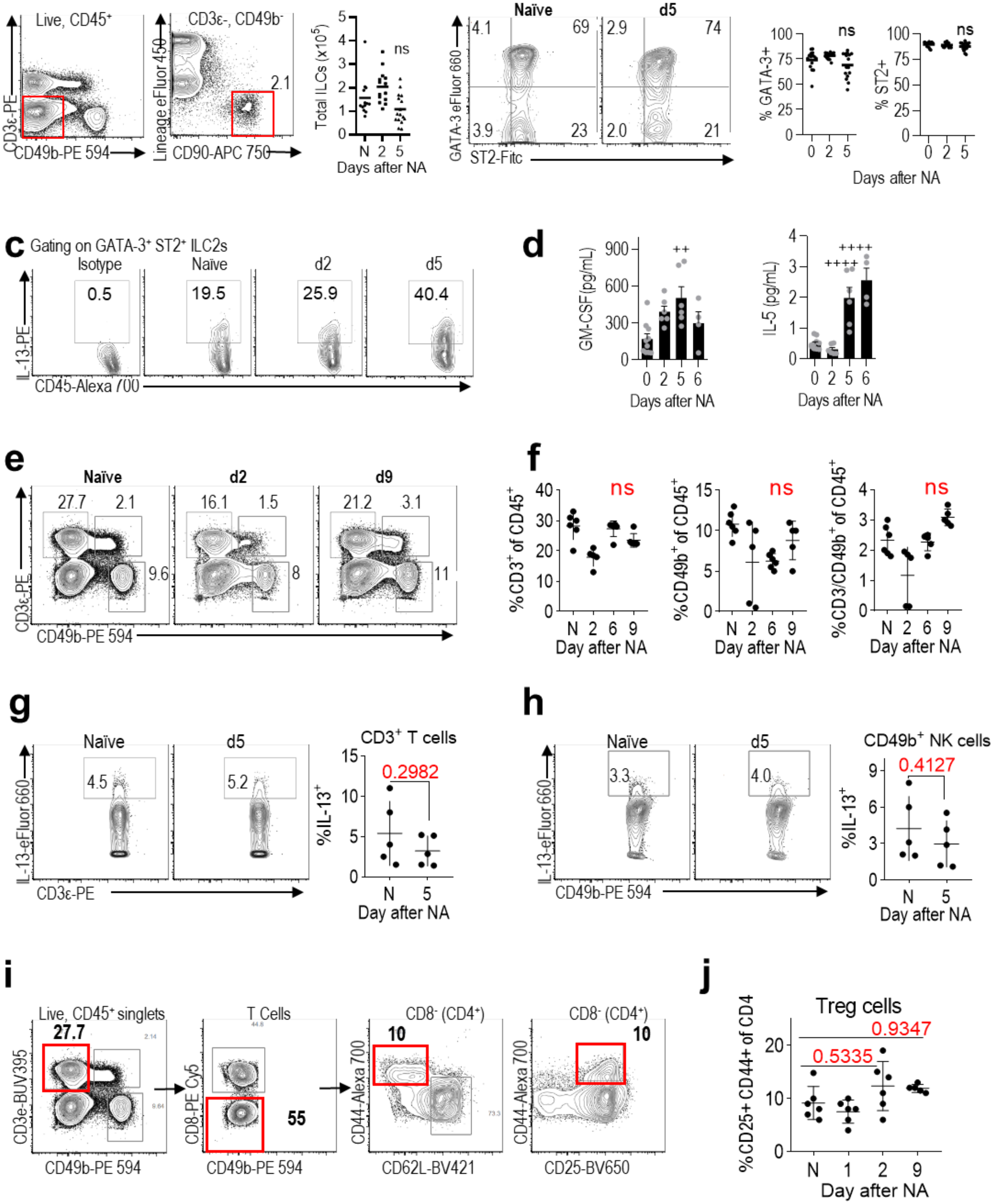
Live, CD45+
CD3ε-PE
CD49b-PE 594
CD3ε-, CD49b-
Lineage eFluor 450
CD90-APC 750
2.1
Total ILCs (x10^5)
ns
N 2 5
Days after NA
Naïve
d5
GATA-3 eFluor 660
ST2-Fitc
4.1
69
3.9
23
2.9
74
2.0
21
% GATA-3+
% ST2+
ns
0 2 5
Days after NA
c
Gating on GATA-3+ ST2+ ILC2s
Isotype
Naïve
d2
d5
0.5
19.5
25.9
40.4
IL-13-PE
CD45-Alexa 700
d
GM-CSF (pg/mL)
IL-5 (pg/mL)
++
++++
++++
0 2 5 6
Days after NA
e
Naïve
d2
d9
27.7
2.1
9.6
16.1
1.5
8
21.2
3.1
11
CD3ε-PE
CD49b-PE 594
f
%CD3+ of CD45+
%CD49b+ of CD45+
%CD3/CD49b+ of CD45+
ns
N 2 6 9
Day after NA
g
Naïve
d5
4.5
5.2
IL-13-eFluor660
CD3ε-PE
CD3+ T cells
0.2982
%IL-13+
N 5
Day after NA
h
Naïve
d5
3.3
4.0
IL-13-eFluor660
CD49b-PE 594
CD49b+ NK cells
0.4127
%IL-13+
N 5
Day after NA
i
Live, CD45+ singlets
27.7
CD3e-BUV395
CD49b-PE 594
T Cells
55
CD8-PE Cy5
CD49b-PE 594
CD8- (CD4+)
10
CD44-Alexa 700
CD62L-BV421
CD8- (CD4+)
10
CD44-Alexa 700
CD25-BV650
j
Treg cells
0.9347
0.5335
%CD25+ CD44+ of CD4
N 1 2 9
Day after NA

Supplementary Figure 6. Identification of ILC2s as the main source of IL-13 in the airways of NA-treated mice.

(a) Gating strategy for quantifying ILCs as live, $CD45^+$, $CD3\varepsilon^-$, $CD49b^-$, $lineage^-$, $CD90^+$ cells, used in Figure 9a left panel. Red boxes denote gating from parent populations. In the right panel, quantification of the total ILCs number in lungs at days 0, 2, and 5 post-NA treatment. **(b)** FACS analysis of ST2 and GATA-3 expression on ILCs as gated in (a). Number in corners of plots denotes percentage for each quadrant of the parent population. The frequency of GATA-3^+ and $ST2^+$ ILCs are quantified in the graphs on the right and Figure 9a right panel. **(c)** ILCs were isolated from the lungs of mice on days 0, 2, 5, and 6 post-NA treatment and stimulated *ex vivo*. Staining of stimulated ILCs for IL-13 production, quantified in Figure 9b. **(d)** GM-CSF and IL-5 quantification in the supernatant of stimulated ILCs as determined by ELISA. **(e-f)** T and NK cell numbers remain unchanged within the lungs of naïve (N) and NA-treated mice, analyzed at the indicated timepoints. (e) Representative plots showing T cells (upper left gate), NK cells (lower right gate), and NKT cell (upper right gate) frequencies as percentages of total viable, $CD45^+$ cells in the lungs. (f) Quantification of each gated population in e. **(g-h)** Whole lung homogenates were stimulated for 4 hours with PMA, ionomycin, and brefeldin A and the percentage of IL-13^+ T cells (g) and NK cells (h) was determined. **(i-j)** Treg cell numbers remain unchanged in the lungs of NA-treated mice, gated as $CD25^+$ $CD44^+$ $CD4^+$ cells. (i) Representative plots showing Treg cell frequencies as percentages of total viable, $CD4^+$ cells in the lungs. (j) Quantification of the Treg population as gated in (i). Data from n=13 (a,b), 4-6 (d), 6 (f), 5 (g-h) and 6 (j) mice, show mean ± SEM pooled from three independent experiments. $^{++}P < 0.01$ and $^{++++}P < 0.0001$ between NA-treated and naïve WT mice using One-Way ANOVA, Bonferroni post-test (a,d and j). Kruskal-Wallis test is used in (b), and Student's T-test in (f,g and h). ns = not significant.

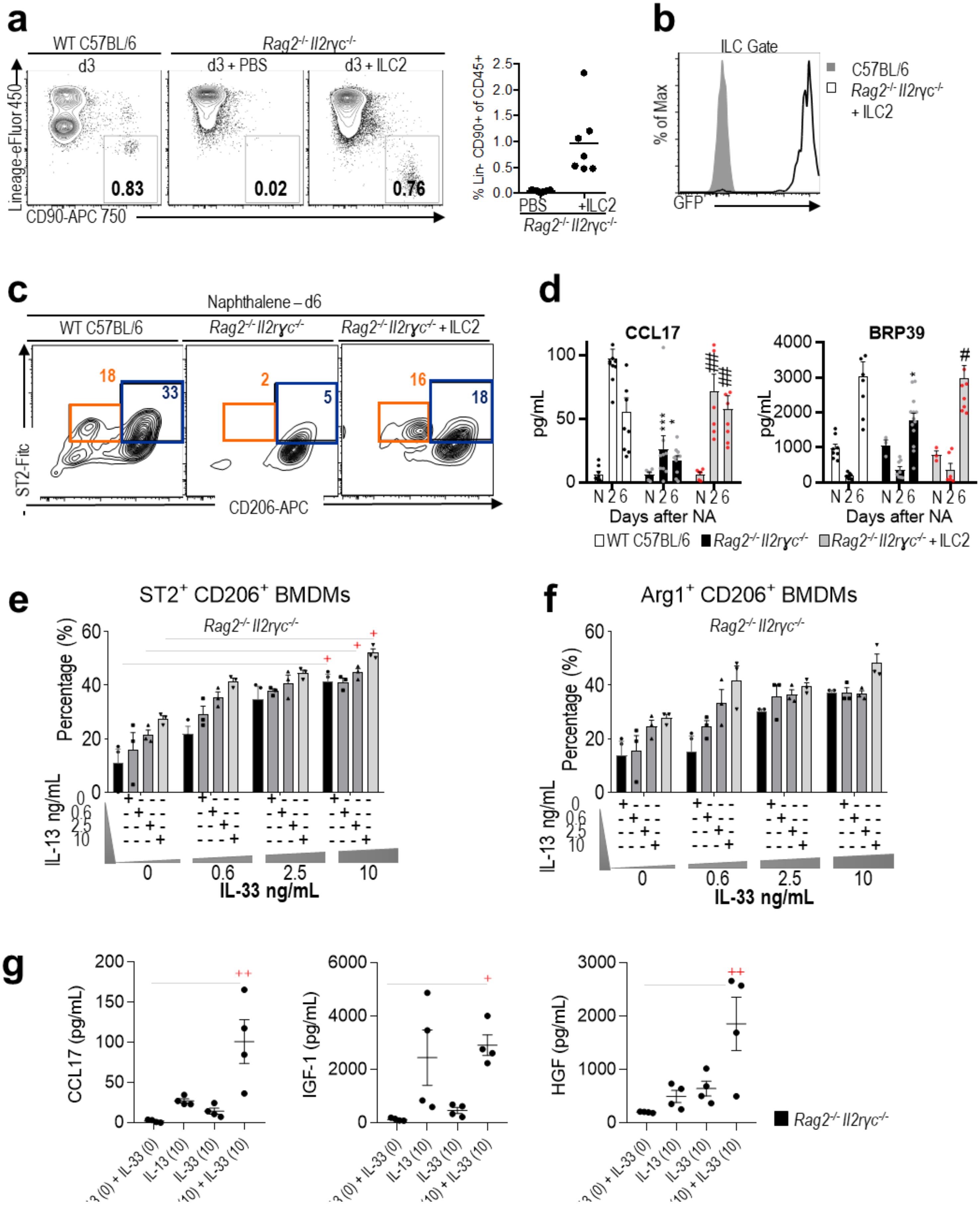
a
WT C57BL/6
Rag2-/- Il2rγc-/-
d3
d3 + PBS
d3 + ILC2
Lineage-eFluor450
CD90-APC 750
0.83
0.02
0.76
% Lin- CD90+ of CD45+
PBS
+ILC2
Rag2-/- Il2rγc-/-
b
ILC Gate
% of Max
GFP
C57BL/6
Rag2-/- Il2rγc-/- + ILC2
c
Naphthalene – d6
WT C57BL/6
Rag2-/- Il2rγc-/-
Rag2-/- Il2rγc-/- + ILC2
18
33
2
5
16
18
ST2-Fitc
CD206-APC
d
CCL17
BRP39
pg/mL
N 2 6
Days after NA
WT C57BL/6
Rag2-/- Il2rγc-/-
Rag2-/- Il2rγc-/- + ILC2
e
ST2+ CD206+ BMDMs
Rag2-/- Il2rγc-/-
Percentage (%)
IL-13 ng/mL
IL-33 ng/mL
f
Arg1+ CD206+ BMDMs
Rag2-/- Il2rγc-/-
Percentage (%)
IL-13 ng/mL
IL-33 ng/mL
g
CCL17 (pg/mL)
IGF-1 (pg/mL)
HGF (pg/mL)
IL-13 (10)
IL-33 (10)
Rag2-/- Il2rγc-/-

Supplementary Figure 7. BMDMs from *Rag2$^{-/-}$/Il2rγc$^{-/-}$* (ILC-deficient) mice differentiate into functional AAMs upon IL-33 treatment.

(a) Representative flow plots showing the frequency as percentage of lineage^{-}, CD90^{+} innate lymphoid cells, (ILCs), in WT C57BL/6, *Rag2$^{-/-}$/Il2rγc$^{-/-}$,* or *Rag2$^{-/-}$/Il2rγc$^{-/-}$* mice that adoptively received GFP^{+} ILC2s, 3d post-NA and quantified on the graph in the right. **(b)** Representative histogram showing GFP expression in WT C57BL/6 or *Rag2$^{-/-}$/Il2rγc$^{-/-}$* mice that adoptively received GFP^{+} ILC2s (*Rag2$^{-/-}$/Il2rγc$^{-/-}$* + ILC2). Graph shows individual mice, where the mean is indicated by a line. Data are from single independent experiment but are representative of two independent experiments in (a-b). **(c-d)** WT C57BL/6 or *Rag2$^{-/-}$/Il2rγc$^{-/-}$* mice that adoptively received GFP^{+} ILC2s (*Rag2$^{-/-}$/Il2rγc$^{-/-}$* + ILC2) were treated with NA and BAL samples were collected at days 0, 2, and 6. (c) Representative flow plots showing the frequency as percentage of ST2^{+} P2 cells in BAL and quantified in Figure 9h. Blue gates and numbers denote frequency of ST2^{+} P3 AAMs. Orange gates and numbers denote frequency of ST2^{+} P2 cells. (d) Quantification of CCL17 and BRP-39 in BAL supernatants. **(e-g)** Bone marrow-derived cells from *Rag2$^{-/-}$/Il2rγc$^{-/-}$,*(C57BL/6 background), mice were cultured in M-CSF alone, (unstimulated), or cultured in the presence of M-CSF with varying concentrations of IL-13 and/or IL-33 for 6 days *ex vivo*. Quantification by FACS of ST2 expression (e) and Arg-1^{+} CD206^{+} (f) BMDMs from *Rag2$^{-/-}$ Il2rγc$^{-/-}$* mice. Quantification of CCL17, IGF-I and HGF from the supernatants of *Rag2$^{-/-}$ Il2rγc$^{-/-}$* BMDMs, stimulated with 10ng/mL IL-13 or 10ng/mL IL-33 or both (g). Bar graphs from n=6 (d), 3 (e,f) and 4 (g) mice, show mean ± SEM pooled from three independent experiments. *$P < 0.05$ between NA-treated *Rag2$^{-/-}$/Il2rγc$^{-/-}$* and WT, $^{\#}$ $P < 0.05$ and $^{\#\#}$ $P < 0.01$ between ILC2 adoptively-transferred and NA-treated *Rag2$^{-/-}$/Il2rγc$^{-/-}$* mice using One-Way ANOVA, Bonferroni post-test (d). $^{+}P < 0.05$ and $^{++}P < 0.~01$ between IL-13 or/and IL-33-stimulated *Rag2$^{-/-}$/Il2rγc$^{-/-}$* at indicated conditions using One-Way (d and g) or Two-Way (e) ANOVA, Bonferroni post-test.

Supplementary Tables

Supplementary Table 1: Immunohistochemistry, Immunofluorescence and Antibodies

Antigen	Host, Isotype	Clone	Dilution	Catalog number	Vendor
F4/80	Rat IgG2b	CL:A3-1	1:200	ab6640	abcam
CCSP	Rabbit	Serum	1:5000	WRAB-3950	Seven Hills
CC10	Goat IgG	Polyclonal	1:500	Sc-9772	Santa Cruz
Ki67-APC	Rat IgG2a	SolA15	1:200	17-5698-82	eBioscience
IL-33	Goat IgG	Polyclonal	1:200	AF3626	R&D systems
ß-tubulin-Fitc	Mouse IgG1	TUB 2.1	1:200	ab11310	abcam
CD3ε-biotin	Hamster IgG	145-2C11	25µg per mL	100304	Biolegend
CD19-biotin	Rat IgG2a	1D3	25µg per mL	553784	BD biosciences
B220-biotin	Rat IgG2a	RA3-6B2	25µg per mL	103204	Biolegend
CD5-biotin	Rat IgG2a	53-7.3	25µg per mL	100604	Biolegend
TCRß-biotin	Hamster IgG	H57-597	25µg per mL	109204	Biolegend
TCRγδ-biotin	Hamster IgG	GL3	25µg per mL	118103	Biolegend
CD11c-biotin	Hamster IgG	N418	25µg per mL	117304	Biolegend
F4/80-biotin	Rat IgG2a	BM8	25µg per mL	123106	Biolegend
Gr-1-biotin	Rat IgG2b	RB6-8C5	25µg per mL	108404	Biolegend
Ter119-biotin	Rat IgG2b	TER-119	25µg per mL	116204	Biolegend
CD49b-biotin	Rat IgM	DX5	25µg per mL	108904	Biolegend
NK1.1-biotin	Mouse IgG2a	PK136	25µg per mL	108704	Biolegend
CD27-biotin	Hamster IgG	LG3A10	25µg per mL	124206	Biolegend
Secondary anti-Goat IgG-Alexa Fluor 488	Donkey IgG	Polyclonal	1:500	A32814	ThermoFisher
Secondary anti-Goat IgG-Alexa Fluor 555	Donkey IgG	Polyclonal	1:500	A32816	ThermoFisher
Secondary anti-Rabbit IgG-Alexa Fluor 488	Donkey IgG	Polyclonal	1:500	A32790	ThermoFisher
Secondary anti-Rabbit IgG-Alexa Fluor 555	Donkey IgG	Polyclonal	1:500	A32794	ThermoFisher

Supplementary Table 2: FACS Antibodies

Antibody	Host, Isotype	Clone	Dilution	Catalog numbe	Vendor
CD45-BUV395	Rat IgG2b	30-F11	1:200	564279	BD Bioscience
F4/80-APC Cy7	Rat IgG2a	BM8	1:50	123118	Biolegend
CD11c-APC	Hamster IgG1	HL3	1:200	550261	BD Bioscience
CD11b-BV421	Rat IgG2b	M1/70	1:200	101251	Biolegend
CD11b-PE	Rat IgG2b	M1/70	1:200	101208	Biolegend
Ly6C-PE CY7	Rat IgM	AL-21	1:200	560593	BD Bioscience
CD64-Alexa Fluor 594	Rat IgG2a	290322	5µL per 10^6 cells	FAB20741P	R&D systems
Ki67-APC	Rat IgG2a	SolA15	1:200	17-5698-82	eBioscience
Arg-1-PE	Sheep IgG	Polyclonal	5µL per 10^6 cells	IC5868P	R&D systems
Biotinylated CD206	Goat IgG	Polyclonal	5µg per mL	BAF2535	R&D systems
CD206-BV650	Rat IgG2a	C068C2	1:200	141723	Biolegend
Biotinylated YM1	Goat IgG	Polyclonal	1:200	BAF2446	R&D systems
Biotinylated FIZZ-1	Goat IgG	Polyclonal	1:200	BAF1523	R&D systems
Siglec-F-BV711	Rat IgG2a	E50-2440	1:200	740764	BD Bioscience
ST2-Fitc	Rat IgG1	DJ8	1:200	101001F	MD Biosciences
Streptavidin-BV421	-	-	1:1000	405226	Biolegend
CD3ε-PE	Hamster IgG1	145-2C11	1:100	553063	BD bioscience
CD3ε- BUV395	Hamster IgG1	145-2C11	1:100	563565	BD bioscience
CD49b-PE/Dazzle 594	Rat IgM	DX5	1:200	108924	Biolegend
CD45-Alexa Fluor 700	Rat IgG2b	30-F11	1:400	103128	Biolegend
CD45-BV510	Rat IgG2b	30-F11	1:300	103137	Biolegend
CD25-BV650	Rat IgG1	PC61	1:400	102037	Biolegend
CD90.2-APC eFluor 780	Rat IgG2a	53-2.1	1:400	47-0902	eBioscience
CD44-Alexa 700	Rat IgG2b	IM7	1:400	103026	Biolegend
CD4-BV750	Rat IgG2b	GK1.5	1:200	100467	Biolegend
CD8-PE Cy5	Rat IgG2a	56-6.7	1:200	100710	Biolegend
CD62L-BV421	Rat IgG2a	MEL-14	1:200	104435	Biolegend
IL-18Rα-PerCP eFluor 710	Rat IgG2a	P3TUNYA	1:100	46-5183	eBioscience
CD90-eFluor 450/BV421	Mouse IgG2a	53-2.1	1:200	48-0900-82	eBioscience
CD45-eFluor 450/BV421	Rat IgG2b	30-F11	1:200	48-0451-82	eBioscience
CD3ε-eFluor 450/BV421	Hamster IgG1	145-2C11	1:200	562600	BD Bioscience
TCRß-eFluor 450/BV421	Hamster IgG1	H57-597	1:200	48-5961-82	eBioscience
TCRγδ-eFluor450/BV421	Hamster IgG1	GL3	1:200	118120	Biolegend
CD5-eFluor 450/BV421	Rat IgG2a	53-7.3	1:200	100617	Biolegend
F4/80-eFluor 450/BV421	Rat IgG2a	BM8	1:200	123131	Biolegend

CD11c-eFluor 450/BV421	Hamster IgG	N418	1:200	117329	Biolegend
Gr-1-eFluor 450/BV421	Rat IgG2b	RB6-8C5	1:200	108433	Biolegend
CD19-eFluor 450/BV421	Rat IgG2a	1D3	1:200	562701	BD Bioscience
FCεRI-eFluor450/BV421	Hamster IgG	MAR-1	1:200	48-5898-82	eBioscience
B220-eFluor 450/BV421	Rat IgG2a	RA3-6B2	1:200	103239	Biolegend
NK1.1-eFluor 450/BV421	Mouse IgG2a	PK136	1:200	108731	Biolegend
CD27-eFluor 450/BV421	Hamster IgG	LG3A10	1:200	561245	BD Bioscience
FoxP3-Alexa Fluor 647	Rat IgG2b	MF23	1:200	560401	BD Bioscience
GATA-3-eFluor 660	Rat IgG2b	TWAJ	1:400	50-9966-42	eBioscience
IL-13-PE	Rat IgG1	clone 13A	1:200	12-7133-41	eBioscience
IL-13- eFluor 660	Rat IgG1	clone 13A	1:200	50-7133-82	eBioscience

Supplementary Table 3: Taqman primers

Retnla (Mm00445109_m1)	*Chil3* (Mm00657889_mH)
Il1rl1 (Mm00516117-m1)	*Itgax* (Mm00498698_m1)
Scgb1a1 (Mm00442046_m1)	*Mrc1/Cd206* (Mm00485148_m1)
Arg1 (Mm00475988_m1)	*Gapdh (*4352932E)
Api5 (m00516117-m1)	*Il1rap* (Mm00492638_m1)
Arg1 (Mm00475988_m1)	*Il1rl1* (Mm00516117_m1)
Bcl-xl (Mm00498698_m1)	*Irf4* (Mm00516431_m1)
Ccnd1 (Mm00657889_mH)	*Klf2* (Mm00500486_m1)
Cd206 (Mm00485148_m1)	*Klf4* (Mm00516104_m1)
c-Mafb (Mm00485148_m1)	*Lrxa* (Mm00443451_m1)
c-Myc (Mm00442046_m1)	*Nos2* (Mm00440502_m1)
Cxcl10 (m00445235_m1)	*P27* (Mm00438168_m1)
E2f1 (Mm00475988_m1)	*P53* (Mm01731290_g1)
Egr1 (Mm00475988_m1)	*Retnla* (Mm00445109_m1)
Gfi1 (Mm00515853_m1)	*Runx3* (Mm00490666_m1)
Hgf (Mm01135184_m1)	*Siglecf* (Mm00556586_m1)
Ifr8 (Mm00492567_m1)	*Stat1* (Mm00439531_m1)
Igf1 (Mm00439560_m1)	*Stat3* (Mm01219775_m1)
Il1a (Mm00439620_m1)	*Stat6* (Mm01160477_m1)
Il1b (Mm00434228_m1)	*Tlr4* (Mm00445274_m1)
Il6 (Mm00446190_m1)	*Tnfa* (Mm00443258_m1)
Gapdh (Mm99999915_g1)	

Supplementary Table 4: Summary of tables for RNA seq data from WT mice

Supplementary data 1: Group comparison result for WT, P2 vs P1
Supplementary data 2: Group comparison result for WT, P3 vs P1
Supplementary data 3: Group comparison result for WT, P3 vs P2
Supplementary data 4: Group comparison result for WT, P3 d6 vs d0
Supplementary data 5: Representative differentially expressed genes between P1, P2 and P3 in each cluster as heatmap of Figure 3b
Supplementary data 6: Summary for Geneset enrichment analysis (Figure 3b, 3d)
Supplementary data 7: Group comparison result (P2 vs P3) for the complete list for Growth Factors, ECM and inflammation (Figure 3c)

Supplementary Table 5: Summary of tables for RNA seq data from ST2KO versus WT mice

Supplementary data 8: Group comparison result for P1, ST2KO vs WT
Supplementary data 9: Group comparison result for P2, ST2KO vs WT
Supplementary data 10: Group comparison result for P3, ST2KO vs WT
Supplementary data 11: Summary for pathway enrichment analysis P2, ST2KO vs WT (Figure 6b)
Supplementary data 12: Summary for pathway enrichment analysis P3, ST2KO vs WT (Figure 6c)
Supplementary data 13: Group comparison result for the complete list for Growth Factors, ECM and immune response, P2 ST2KO vs WT (Figure 6d)
Supplementary data 14: Group comparison result for the complete list for Growth Factors, ECM and immune response, P3 ST2KO vs WT (Figure 6d)

Supplementary Table 6: Statistical analyses methods

	**TEST USED**		***n***			**DESCRIPTIVE STATS (AVERAGE, VARIANCE)**		***P* VALUE**	
**FIGURE #**	**WHICH TEST**	**SECTION & PARAGRAPH**	**EXACT VALUE**	**DEFINED**	**SECTION & PARAGRAPH**	**REPORTED**	**SECTION & PARAGRAPH**	**EXACT VALUE**	**SECTION & PARAGRAPH**
Fig 1b	One-Way ANOVA, Bonferroni post-test	Methods	8 per group	Lung sections from individual mice C57BL6	Figure Legend	Error bars represent Mean ± SEM	Figure Legend	d2-d15; <0.0001<br>d21; 0.0312	Methods
Fig 1d	One-Way ANOVA, Bonferroni post-test	Methods	9 per group	BAL cells from individual mice C57BL6	Figure Legend	Error bars represent Mean ± SEM	Figure Legend	d2, 0.0322;<br>d3, 0.0008;<br>d6, <0.0001;<br>d9, <0.0001;<br>d15, <0.0001;<br>d21, 0.0065	Methods
Fig 1e	One-Way ANOVA, Bonferroni post-test	Methods	For all cytokines 8 per group	BAL supernatants from individual mice C57BL6	Figure Legend	Error bars represent Mean ± SEM	Figure Legend	IL-13 d2, 0.0006;<br>IL-13 d3, 0.0064;<br>IL-13 d6, 0.0298;<br>CCL2 d2, <0.0001;<br>CCL2 d3, 0.0001;<br>CXCL10 d1, 0.0431;<br>CXCL10 d2, <0.0001;<br>CXCL10 d3, 0.0019;<br>IL-1α d1, <0.0001;<br>IL-1α d2, 0.0276	Methods
Fig 1g	One-Way ANOVA, Bonferroni post-test	Methods	6 per group	BAL cells from individual mice C57BL6	Figure Legend	Error bars represent Mean ± SEM	Figure Legend	P1 d6, 0.0031;<br>P1 d9, <0.0001;<br>P1 d15, 0.0036;<br>P2 d6, 0.0166;<br>P2 d9, <0.0001;<br>P2 d15, <0.0001;<br>P2 d21, 0.0014;<br>P3 d6, <0.0001;<br>P3 d9, <0.0001;<br>P3 d15, <0.0001;<br>P3 d21, 0.0027	Methods
Fig 1i	One-Way ANOVA, Bonferroni post-test	Methods	6 per group	BAL cells from individual mice C57BL6	Figure Legend	Error bars represent Mean ± SEM	Figure Legend	Ki-67 (P2+P3) d3, 0.0163;<br>Ki-67 (P2+P3) d6, <0.0001;<br>Ki-67 (P2+P3) d9, <0.0001;<br>Ki-67 (P2+P3) d15, <0.0001;<br>Ki-67 (P2+P3) d21, 0.0003	Methods
Fig 2b	One-Way ANOVA, Bonferroni post-test	Methods	8 per group	Lung mRNAs from individual mice C57BL6	Figure Legend	Error bars represent Mean ± SEM	Figure Legend	Depleted d21, 0.0026;<br>Depleted d35, <0.0001;<br>Transferred d21, 0.0229;<br>Transferred d35, <0.0001	Methods

Fig 2c	One-Way ANOVA, Bonferroni post-test	Methods	3 per group	Lung sections from individual mice C57BL6	Figure Legend	Error bars represent Mean ± SEM	Figure Legend	Depleted d21, 0.0016;<br>Depleted d35, <0.0001;<br>Transferred d21, 0.0021;<br>Transferred d35, <0.0001	Methods
Fig 2d	One-Way ANOVA, Bonferroni post-test	Methods	6 per group	BAL cells from individual mice C57BL6	Figure Legend	Error bars represent Mean ± SEM	Figure Legend	P2 d6, 0.0428;<br>P2 d9, <0.0001;<br>P2 d15, <0.0001;<br>P2 d21, 0.0134;<br>P3 d6, <0.0001;<br>P3 d9, <0.0001;<br>P3 d15, <0.0001;<br>P3 d21,0.0011	Methods
Fig 4a	One-Way ANOVA, Bonferroni post-test	Methods	7 per group	BAL cells mRNAs from individual mice C57BL6	Figure Legend	Error bars represent Mean ± SEM	Figure Legend	d6, 0.0487;<br>d9, 0.0001	Methods
Fig 4e	One-Way ANOVA, Bonferroni post-test	Methods	8 per group	BAL supernatants from individual mice C57BL6	Figure Legend	Error bars represent Mean ± SEM	Figure Legend	sST2, d1, 0.0283;<br>sST2, d2, <0.0001;<br>sST2, d3, 0.0019	Methods
Fig 4f	One-Way ANOVA, Bonferroni post-test	Methods	9 per group	Whole lung homogenates from individual mice C57BL6	Figure Legend	Error bars represent Mean ± SEM	Figure Legend	IL-33, d2, 0.0028;<br>IL-33, d3, <0.0001;<br>IL-33, d6, <0.0001;<br>IL-33, d9, <0.0001;<br>IL-33, d15, <0.0001;<br>IL-33, d21, 0.0385	Methods
Fig 5b	One-Way ANOVA, Bonferroni post-test	Methods	10 per group	Lung sections from individual *ST2*$^{-/-}$ *vs* C57BL6 mice	Figure Legend	Error bars represent Mean ± SEM	Figure Legend	d21, 0.0439;<br>d35, <0.0001	Methods
Fig 5c	Two-Way ANOVA, Bonferroni post-test	Methods	5 per group	BAL cells from individual *ST2*$^{-/-}$ *vs* C57BL6 mice	Figure Legend	Error bars represent Mean ± SEM	Figure Legend	P1 d6, <0.0001;<br>P1 d9, <0.0001;<br><br>P2 d9, <0.0001;<br><br>P3 d2, 0.0003;<br>P3 d6, <0.0001;<br>P3 d9, <0.0001;<br>P3 d21, <0.0001	Methods
Fig 5d	Two-Way ANOVA, Bonferroni post-test	Methods	N (8)<br>d1 (8)<br>d2 (8)<br>d3(6)<br>d6 (8)	BAL supernatants from individual *ST2*$^{-/-}$ *vs* C57BL6 mice	Figure Legend	Error bars represent Mean ± SEM	Figure Legend	IL-13 d1, 0.0078;<br>IL-13 d2, <0.0001;<br>IL-13 d3, 0.0920	Methods
Fig 5d	Two-Way ANOVA, Bonferroni post-test	Methods	5 per group	BAL supernatants from individual *ST2*$^{-/-}$ *vs* C57BL6 mice	Figure Legend	Error bars represent Mean ± SEM	Figure Legend	CCL2 d1, >0.9999;<br>CCL2 d2, >0.9999;<br>CCL2 d3, 0.0423	Methods

Fig 5e	One-Way ANOVA, Bonferroni post-test	Methods	For all cytokines 5 per group	BAL cell lysates from individual *ST2*$^{-/-}$ *vs* C57BL6 mice	Figure Legend	Error bars represent Mean ± SEM	Figure Legend	IGF-1 d3, 0.0235;<br>IGF-1 d6, 0.0492;<br>HGF d3, 0.0019;<br>HGF d6, 0.0036	Methods
Fig 5f	One-Way ANOVA, Bonferroni post-test	Methods	5 per group	BAL cells from individual *ST2*$^{-/-}$ *vs* C57BL6 mice	Figure Legend	Error bars represent Mean ± SEM	Figure Legend	Ki67 d9, <0.0001	Methods
Fig 5h	One-Way ANOVA, Bonferroni post-test	Methods	10 per group	Lung sections from individual *ST2*$^{-/-}$, AAM transferred *ST2*$^{-/-}$ vs C57BL6 mice	Figure Legend	Error bars represent Mean ± SEM	Figure Legend	ST2$^{-/-}$ *vs* WT d35, 0.0031;<br><br>AAM transferred ST2$^{-/-}$ *vs* ST2$^{-/-}$ d35, 0.0031	Methods
Fig 5i	One-Way ANOVA, Bonferroni post-test	Methods	8 per group	Lung mRNAs from individual *ST2*$^{-/-}$ *vs* WT C57BL6 mice	Figure Legend	Error bars represent Mean ± SEM	Figure Legend	d35, ST2$^{-/-}$ *vs* WT, 0.0029;<br>d35, AAM transferred into ST2$^{-/-}$ *vs* WT, 0.0031	Methods
Fig 7b	Two-Way ANOVA, Bonferroni post-test	Methods	3 per group	BMDMs from individual *ST2*$^{-/-}$ *vs* WT C57BL6 mice	Figure Legend	Error bars represent Mean ± SEM	Figure Legend	G0/G1, WT, IL33 (0.6) *vs* unstimulated, 0.0133;<br>G0/G1, WT, IL33 (2.5) *vs* unstimulated, <0.0001;<br>G0/G1, WT, IL33 (10) *vs* unstimulated, <0.0001;<br>G0/G1, IL33 (0.6), ST2$^{-/-}$ *vs* WT, <0.0001;<br>G0/G1, IL33 (2.5), ST2$^{-/-}$ *vs* WT, <0.0001;<br>G0/G1, IL33 (10), ST2$^{-/-}$ *vs* WT, <0.0001;<br><br>S, WT, IL33 (0.6) *vs* unstimulated, 0.0015;<br>S, WT, IL33 (2.5) *vs* unstimulated, <0.0001;<br>S, WT, IL33 (10) *vs* unstimulated, <0.0001;<br>S, IL33 (0.6), ST2$^{-/-}$ *vs* WT, <0.0001;<br>S, IL33 (2.5), ST2$^{-/-}$ *vs* WT, <0.0001;<br>S, IL33 (10), ST2$^{-/-}$ *vs* WT, <0.0001;	Methods
Fig 8b	Two-Way ANOVA, Bonferroni post-test	Methods	4 per group	BMDM cells from individual GFP ST2 mice	Figure Legend	Error bars represent Mean ± SEM	Figure Legend	GFP ST2, IL-13, 0.0270;<br>GFP ST2, IL-33, 0.0019;<br>GFP ST2, IL-13 (10) IL33 (10), <0.0001<br>GFP ST2, IL-13 (10) IL33 (10 *vs* IL-33, 0.0198	Methods
Fig 8d	Two-Way ANOVA, Bonferroni post-test	Methods	3 per group	BMDM cells from individual *ST2*$^{-/-}$ *vs* WT C57BL6 mice	Figure Legend	Error bars represent Mean ± SEM	Figure Legend	CD206 Arg1, IL-13 (10), 0.0025;<br>CD206 Arg1, IL-33 (10), 0.0017;<br>CD206 Arg1, IL-13 (10) IL33 (10), <0.0001	Methods
Fig 8e	Two-Way ANOVA, Bonferroni post-test	Methods	3 per group	BMDM supernatants from individual *ST2*$^{-/-}$ *vs* WT C57BL6 mice	Figure Legend	Error bars represent Mean ± SEM	Figure Legend	HGF, WT, IL33 (2.5) *vs* unstimulated, 0.0350;<br>HGF, WT, IL33 (2.5) IL13 (10) *vs* unstimulated, 0.0450;<br>HGF, WT, IL33 (10) *vs* unstimulated, <0.0001;<br>HGF, WT, IL33 (10) IL13 (10) *vs* unstimulated, <0.0001;<br>HGF, IL33 (0), ST2$^{-/-}$ *vs* WT, 0.0055;<br>HGF, IL33 (0.6), ST2$^{-/-}$ *vs* WT, 0.0007;<br>HGF, IL33 (2.5), ST2$^{-/-}$ *vs* WT, <0.0001;<br>HGF, IL33 (10), ST2$^{-/-}$ *vs* WT, <0.0001;<br>HGF, IL33 (0) IL13 (10), ST2$^{-/-}$ *vs* WT, 0.0053 ;<br>HGF, IL33 (0.6) IL13 (10), ST2$^{-/-}$ *vs* WT, 0.0035;<br>HGF, IL33 (2.5) IL13 (10), ST2$^{-/-}$ *vs* WT, 0.0004;<br>HGF, IL33 (10) IL13 (10), ST2$^{-/-}$ *vs* WT, <0.0001;	Methods

Fig 8f	Two-Way ANOVA, Bonferroni post-test	Methods	3 per group	BMDM supernatants from individual *ST2*$^{-/-}$ *vs* WT C57BL6 mice	Figure Legend	Error bars represent Mean ± SEM	Figure Legend	IGF-1, WT, IL33 (10) *vs* unstimulated, 0.0196;<br>IGF-1, WT, IL33 (0) IL13 (10) *vs* unstimulated, <0.0001;<br>IGF-1, WT, IL33 (0.6) IL13 (10) *vs* unstimulated, <0.0001;<br>IGF-1, WT, IL33 (2.5) IL13 (10) *vs* unstimulated, 0.0071;<br>IGF-1, WT, IL33 (10) IL13 (10) *vs* unstimulated, 0.0129;<br>IGF-1, IL33 (0.6), ST2$^{-/-}$ *vs* WT, 0.0327;<br>IGF-1, IL33 (2.5), ST2$^{-/-}$ *vs* WT, 0.0059;<br>IGF-1, IL33 (10), ST2$^{-/-}$ *vs* WT, 0.0496;<br>IGF-1, IL33 (0.6) IL13 (10), ST2$^{-/-}$ *vs* WT, 0.0019	
Fig 8g	Two-Way ANOVA, Bonferroni post-test	Methods	3 per group	BMDM supernatants from individual *ST2*$^{-/-}$ *vs* WT C57BL6 mice	Figure Legend	Error bars represent Mean ± SEM	Figure Legend	BRP39, WT, IL33 (2.5) *vs* unstimulated, <0.0001;<br>BRP39, WT, IL33 (2.5) IL13 (10) *vs* unstimulated, 0.0002;<br>BRP39, WT, IL33 (10) *vs* unstimulated, <0.0001;<br>BRP39, WT, IL33 (2.5) IL13 (10) *vs* unstimulated, <0.0001;<br>BRP39, IL33 (2.5), ST2$^{-/-}$ *vs* WT, <0.0001;<br>BRP39, IL33 (10), ST2$^{-/-}$ *vs* WT, <0.0001;<br>BRP39, IL33 (2.5) IL13 (10), ST2$^{-/-}$ *vs* WT, <0.0001;<br>BRP39, IL33 (10) IL13 (10), ST2$^{-/-}$ *vs* WT, <0.0001;	Methods
Fig 9a	One-Way ANOVA, Bonferroni post-test	Methods	(N) 15<br>(2) 15<br>(5) 16	ILCS from the lungs of Individual mice	Figure legends, methods	Individual mice with mean	Figure Legend	Total ILCs Frequency, 0.0003	Methods
Fig 9a	One-Way ANOVA, Bonferroni post-test	Methods	(N)15<br>(2)15<br>(5)16	ILCS from the lungs of Individual mice	Figure legends, methods	Individual mice with mean	Figure legend	% ST2+ GATA-3+, 0.1326;<br><br>ST2, 0.3734	Methods
Fig 9b	One-Way ANOVA, Bonferroni post-test	Methods	(N) 7<br>(2) 6<br>(5) 6	Pooled ILCs from WT B6 mice stimulated with IL-2 and IL-33	Figure Legend, Methods	Error bars represent Mean ± SEM	Figure legend	% IL-13+, <0.0001	Methods
Fig 9c	One-Way ANOVA, Bonferroni post-test	Methods	For all cytokines<br>(N) 10<br>(2) 6<br>(5) 6<br>(6) 4	Pooled ILCs from WT B6 mice stimulated with IL-2 and IL-33	Figure Legend, Methods	Error bars represent Mean ± SEM	Figure legend	IL-13 d5, <0.0001<br>IL-13 d6, <0.0001	Methods
Fig 9c	One-Way ANOVA, Bonferroni post-test	Methods	For all cytokines<br>(N) 10<br>(2) 6<br>(5) 6<br>(6) 4	Pooled ILCs from WT B6 mice stimulated with IL-2 and IL-33	Figure Legend, Methods	Error bars represent Mean ± SEM	Figure legend	Amphiregulin N vs. 2, > 0.9999;<br>Amphiregulin N vs. 5, > 0.9999;<br>Amphiregulin N vs. d6, > 0.9999;<br>Amphiregulin d2 vs. d5, > 0.9999;<br>Amphiregulin d2 vs. d6, > 0.9999;<br>Amphiregulin d5 vs. d6, > 0.9999;	Methods
Fig 9e	Two-Way ANOVA, Bonferroni post-test	Methods	4 per group	Lung mRNA from individual *Rag2*$^{-/-}$ *Il2rγc*$^{-/-}$, ILC2 transferred *Rag2*$^{-/-}$ *Il2rγc*$^{-/-}$ and C57BL6 mice	Figure Legend	Error bars represent Mean ± SEM	Figure Legend	*Scgb1a1* d35 Rag2$^{-/-}$ Il2rγc$^{-/-}$ *vs* d35 WT, 0.005;<br><br>*Scgb1a1* d35 Rag2$^{-/-}$ Il2rγc$^{-/-}$ *vs* d35 Rag2$^{-/-}$ Il2rγc$^{-/-}$+ILC2, 0.0018	Methods

Fig 9g	One-Way ANOVA, Bonferroni post-test	Methods	5 per group	BAL cells from individual *Rag2*$^{-/-}$ *Il2rγc*$^{-/-}$, ILC2 transferred *Rag2*$^{-/-}$ *Il2rγc*$^{-/-}$ and C57BL6 mice	Figure Legend	Error bars represent Mean ± SEM	Figure Legend	P1 Rag2$^{-/-}$ Il2rγc$^{-/-}$ *vs* WT d6, 0.0359;<br>P1 ILC2 transferred Rag2$^{-/-}$ Il2rγc$^{-/-}$ *vs* Rag2$^{-/-}$ Il2rγc$^{-/-}$ *vs* d6, <0.0001;<br>P2 Rag2$^{-/-}$ Il2rγc$^{-/-}$ *vs* WT d6, <0.0001;<br>P2 ILC2 transferred Rag2$^{-/-}$ Il2rγc$^{-/-}$ *vs* Rag2$^{-/-}$ Il2rγc$^{-/-}$ *vs* d6, 0.0189;<br>P3 Rag2$^{-/-}$ Il2rγc$^{-/-}$ *vs* WT d6, 0.0344;<br>P3 ILC2 transferred Rag2$^{-/-}$ Il2rγc$^{-/-}$ *vs* Rag2$^{-/-}$ Il2rγc$^{-/-}$ *vs* d6, 0.0484	Methods
Fig 9h	One-Way ANOVA, Bonferroni post-test	Methods	5 per group	BAL cells from individual *Rag2*$^{-/-}$ *Il2rγc*$^{-/-}$, ILC2 transferred *Rag2*$^{-/-}$ *Il2rγc*$^{-/-}$ and C57BL6 mice	Figure Legend	Error bars represent Mean ± SEM	Figure Legend	ST2+ P2 Rag2$^{-/-}$ Il2rγc$^{-/-}$ *vs* WT d6, <0.0001;<br><br>ST2+ P2 ILC2 transferred Rag2$^{-/-}$ Il2rγc$^{-/-}$ *vs* Rag2$^{-/-}$ Il2rγc$^{-/-}$ *vs* d6, 0.0004;	Methods
Fig 9i	One-Way ANOVA, Bonferroni post-test	Methods	6 per group	Whole lung homogenates from individual *Rag2*$^{-/-}$ *Il2rγc*$^{-/-}$, ILC2 transferred *Rag2*$^{-/-}$ *Il2rγc*$^{-/-}$ and C57BL6 mice	Figure Legend	Error bars represent Mean ± SEM	Figure Legend	Lung IL-13 Rag2$^{-/-}$ Il2rγc$^{-/-}$ *vs* WT d6, 0.0463;<br><br>Lung IL-13 ILC2 transferred Rag2$^{-/-}$ Il2rγc$^{-/-}$ *vs* Rag2$^{-/-}$ Il2rγc$^{-/-}$ *vs* d6, <0.0001;	Methods
Fig 9j	One-Way ANOVA, Bonferroni post-test	Methods	6 per group	BAL supernatants from individual *Rag2*$^{-/-}$ *Il2rγc*$^{-/-}$, ILC2 transferred *Rag2*$^{-/-}$ *Il2rγc*$^{-/-}$ and C57BL6 mice	Figure Legend	Error bars represent Mean ± SEM	Figure Legend	BAL IL-13 Rag2$^{-/-}$ Il2rγc$^{-/-}$ *vs* WT d2, <0.0001;<br><br>BAL IL-13 Rag2$^{-/-}$ Il2rγc$^{-/-}$ *vs* WT d6, 0.0120;<br><br>BAL IL-13 ILC2 transferred Rag2$^{-/-}$ Il2rγc$^{-/-}$ *vs* Rag2$^{-/-}$ Il2rγc$^{-/-}$ *vs* d6, <0.0001	Methods
Fig 9k	One-Way ANOVA, Bonferroni post-test	Methods	8 per group	BAL supernatants from individual *Rag2*$^{-/-}$ *Il2rγc*$^{-/-}$, ILC2 transferred *Rag2*$^{-/-}$ *Il2rγc*$^{-/-}$ and C57BL6 mice	Figure Legend	Error bars represent Mean ± SEM	Figure Legend	IGF-1 Rag2$^{-/-}$ Il2rγc$^{-/-}$ *vs* WT d2, <0.0001;<br>IGF-1 ILC2 transferred Rag2$^{-/-}$ Il2rγc$^{-/-}$ *vs* Rag2$^{-/-}$ Il2rγc$^{-/-}$ *vs* d2, 0.0389;<br>IGF-1 ILC2 transferred Rag2$^{-/-}$ Il2rγc$^{-/-}$ *vs* Rag2$^{-/-}$ Il2rγc$^{-/-}$ *vs* d6, 0.0002;<br><br>HGF Rag2$^{-/-}$ Il2rγc$^{-/-}$ *vs* WT d2, 0.0223;<br>HGF ILC2 transferred Rag2$^{-/-}$ Il2rγc$^{-/-}$ *vs* Rag2$^{-/-}$ Il2rγc$^{-/-}$ *vs* d2, 0.0288;<br>HGF ILC2 transferred Rag2$^{-/-}$ Il2rγc$^{-/-}$ *vs* Rag2$^{-/-}$ Il2rγc$^{-/-}$ *vs* d6, 0.0053	Methods
Fig S1a	One-Way ANOVA, Bonferroni post-test	Methods	8 per group	Lung mRNAs from individual mice C57BL6	Figure Legend	Error bars represent Mean ± SEM	Figure Legend	d2-d15, <0.0001;<br>d21, 0.0445	Figure Legend
Fig S1e	One-Way ANOVA, Bonferroni post-test	Methods	5 per group	BAL cells from individual depleted *vs* WT C57BL6 mice	Figure Legend	Error bars represent Mean ± SEM	Figure Legend	P1, CD11b, d6, depleted *vs* WT, 0.0005;<br>P1, CD11b, d9, depleted *vs* WT, <0.0001;<br>P1, CD11b, d15, depleted *vs* WT, 0.0015;<br>P1, YM1, d6, depleted *vs* WT, 0.0220;<br>P1, YM1, d9, depleted *vs* WT, <0.0001;<br>P2, CD11b, d9, depleted *vs* WT, 0.0258;<br>P2, CD11b, d9, depleted *vs* WT, <0.0001;<br>P2, CD11b, d15, depleted *vs* WT, <0.0001;	Methods

								P2, CD11b, d21, depleted *vs* WT, 0.0001;<br>P2, CD11c, d9, depleted *vs* WT, <0.0001;<br>P2, CD11c, d15, depleted *vs* WT, <0.0001;<br>P2, CD11c, d21, depleted *vs* WT, 0.0016;<br>P2, Arg-1, d6, depleted *vs* WT, 0.0469;<br>P2, Arg-1, d9, depleted *vs* WT, <0.0001;<br>P2, Arg-1, d15, depleted *vs* WT, <0.0001;<br>P2, Arg-1, d21, depleted *vs* WT, 0.0002;<br>P3, CD206, d6, depleted *vs* WT, <0.0001;<br>P3, CD206, d9, depleted *vs* WT, <0.0001;<br>P3, CD206, d15, depleted *vs* WT, <0.0001;<br>P3, CD206, d21, depleted *vs* WT, <0.0001;<br>P3, YM1, d6, depleted *vs* WT, <0.0001;<br>P3, YM1, d9, depleted *vs* WT, <0.0001;<br>P3, YM1, d15, depleted *vs* WT, <0.0001;<br>P3, YM1, d21, depleted *vs* WT, 0.0186;<br>P3, Arg-1, d6, depleted *vs* WT, <0.0001;<br>P3, Arg-1, d9, depleted *vs* WT, <0.0001;<br>P3, Arg-1, d15, depleted *vs* WT, 0.0003;<br>P3, FIZZ-1, d6, depleted *vs* WT, <0.0001;<br>P3, FIZZ-1, d9, depleted *vs* WT, <0.0001;<br>P3, FIZZ-1, d15, depleted *vs* WT, <0.0001;<br>P3, FIZZ-1, d21, depleted *vs* WT, 0.0417	
Fig S1f	One-Way ANOVA, Bonferroni post-test	Methods	8 per group	BAL cell mRNAs from individual WT C57BL6 mice	Figure Legend	Error bars represent Mean ± SEM	Figure Legend	*Itgax*, d6, 0.0010;<br>*Itgax*, d9, 0.0006;<br>*Cd206*, d6, 0.0145;<br>*Cd206*, d9, 0.0009;<br>*Retnla*, d9, 0.0003;<br>*Retnla*, d15, 0.0079;<br>*Arg1*, d3, <0.0001;<br>*Arg1*, d6, <0.0001;<br>*Arg1*, d9, 0.0074;<br>*Chil3*, d3, 0.0431;<br>*Chil3*, d6, 0.0015;	Methods
Fig S2a	Two-Way ANOVA, Bonferroni post-test	Methods	4 per group	BAL cell from individual depleted *vs* WT C57BL6 mice	Figure Legend	Error bars represent Mean ± SEM	Figure Legend	d3, depleted *vs* WT, 0.0355;<br>d6, depleted *vs* WT, <0.0001;<br>d9, depleted *vs* WT, <0.0001;<br>d15, depleted *vs* WT, <0.0001;<br>d21, depleted *vs* WT, 0.0008	Methods
Fig S2b	Two-Way ANOVA, Bonferroni post-test	Methods	8 per group	BAL cell mRNAs from individual depleted *vs* WT C57BL6 mice	Figure Legend	Error bars represent Mean ± SEM	Figure Legend	*Cd206*, d6, depleted *vs* WT, 0.0098;<br>*Cd206*, d9, depleted *vs* WT, 0.0009;<br>*Retnla*, d9, depleted *vs* WT, 0.0013;<br>*Retnla*, d15, depleted *vs* WT, 0.0027;<br>*Arg1*, d3, depleted *vs* WT, <0.0108;<br>*Arg1*, d6, depleted *vs* WT, 0.0008;<br>*Arg1*, d9, depleted *vs* WT, 0.0041;<br>*Chil3*, d3, depleted *vs* WT, 0.0019;<br>*Chil3*, d6, depleted *vs* WT, 0.0361;<br>*Chil3*, d9, depleted *vs* WT, 0.0440	Methods

Fig S4a	One-Way ANOVA, Bonferroni post-test	Methods	8 per group	Lung mRNAs from individual *$ST2^{-/-}$ vs* WT C57BL6 mice	Figure Legend	Error bars represent Mean ± SEM	Figure Legend	d35, $ST2^{-/-}$ *vs* WT, <0.0001	Methods
Fig S4b	One-Way ANOVA, Bonferroni post-test	Methods	8 per group	Lung sections from individual *$ST2^{-/-}$ vs* C57BL6 mice	Figure Legend	Error bars represent Mean ± SEM	Figure Legend	$CCSP^{low}$ d35, <0.0001;<br>$CCSP^{high}$ d35, <0.0001	Methods
Fig S4b	One-Way ANOVA, Bonferroni post-test	Methods	8 per group	Lung sections from individual *$ST2^{-/-}$ vs* C57BL6 mice	Figure Legend	Error bars represent Mean ± SEM	Figure Legend	$CCSP^{low}$ Ki-67^{+} d6, 0.0002;<br>$CCSP^{low}$ Ki-67^{+} d9, 0.0001	Methods
Fig S4d	Two-Way ANOVA, Bonferroni post-test	Methods	6 per group	BAL supernatants from individual *$ST2^{-/-}$ vs* C57BL6 mice	Figure Legend	Error bars represent Mean ± SEM		CXCL10, d3 compared to d3, 0.0345	Methods
Fig S4e	Two-Way ANOVA, Bonferroni post-test	Methods	5 per group	Pooled BAL cell lysates *from $ST2^{-/-}$ vs* WT C57BL6 mice	Figure Legend	Error bars represent Mean	Figure Legend	CXCL12, d9 compared to d0, <0.0001;<br>MCP-5, d9 compared to d0, 0.0097;<br>CCL2, d9 compared to d0, 0.0500;<br>CCL2, d9 compared to d0, 0.0062;<br>CCL6, d2 compared to d0, 0.0097;<br>CCL6, d9 compared to d0, <0.0001<br>IL-16, d2 compared to d0, <0.0001;<br>IL-16, d9 compared to d0, <0.0001;<br><br>d9, CXCL12, $ST2^{-/-}$ *vs* WT, <0.0001;<br>d9, MCP-5, $ST2^{-/-}$ *vs* WT, 0.0017;<br>d2, IL-16, $ST2^{-/-}$ *vs* WT, 0.0492;<br>d9, IL-16, $ST2^{-/-}$ *vs* WT, 0.0017;<br>d9, CCL2, $ST2^{-/-}$ *vs* WT, 0.0100;	Methods
Fig S4f	One-Way ANOVA, Bonferroni post-test	Methods	For all cytokines 5 per group	BAL cell lysates from individual *$ST2^{-/-}$ vs* C57BL6 mice	Figure Legend	Error bars represent Mean ± SEM	Figure Legend	BRP39 d3, <0.0001;<br>BRP39 d6, 0.0392;<br>CCL17 d1, 0.0399;<br>CCL17 d3,0.0261;<br>CCL17 d6, 0.0002;	Methods
Fig S5b	Two-Way ANOVA, Bonferroni post-test	Methods	3 per group	BMDM cells from individual *$ST2^{-/-}$ vs* WT C57BL6 mice	Figure Legend	Error bars represent Mean ± SEM	Figure Legend	G0/G1, WT, IL33 (0.6) *vs* unstimulated, 0.0133;<br>G0/G1, WT, IL33 (2.5) *vs* unstimulated, <0.0001;<br>G0/G1, WT, IL33 (10) *vs* unstimulated, <0.0001;<br>G0/G1, IL33 (0.6), $ST2^{-/-}$ *vs* WT, <0.0001;<br>G0/G1, IL33 (2.5), $ST2^{-/-}$ *vs* WT, <0.0001;<br>G0/G1, IL33 (10), $ST2^{-/-}$ *vs* WT, <0.0001;<br><br>S, WT, IL33 (0.6) *vs* unstimulated, 0.0015;<br>S, WT, IL33 (2.5) *vs* unstimulated, <0.0001;<br>S, WT, IL33 (10) *vs* unstimulated, <0.0001;<br>S, IL33 (0.6), $ST2^{-/-}$ *vs* WT, <0.0001;<br>S, IL33 (2.5), $ST2^{-/-}$ *vs* WT, <0.0001;<br>S, IL33 (10), $ST2^{-/-}$ *vs* WT, <0.0001;	Methods

Fig S6a	One-Way ANOVA, Bonferroni post-test	Methods	(N) 15<br>(2) 15<br>(5) 16	ILCs from the lungs of Individual mice	Methods	Individual mice with mean	Figure Legend	Total ILCs Frequency, 0.0003;<br>Total ILCs num, 0.4595	Methods
Fig S6b	Kruskal-Wallis test	Methods	(N) 14<br>(2) 15<br>(5) 16	Pooled ILCs from WT B6 mice stimulated with IL-2 and IL-33	Methods	Error bars represent Mean ± SEM	Figure legend	%GATA3+, 0.1326;<br><br>%ST2+, 0.3734	Methods
Fig S6d	One-Way ANOVA, Bonferroni post-test	Methods	For all cytokines<br>(N) 10<br>(2) 6<br>(5) 6<br>(6) 4	Pooled ILCs from WT B6 mice stimulated with IL-2 and IL-33	Methods	Error bars represent Mean ± SEM	Figure legend	GM-CSF d2, 0.0828;<br>GM-CSF d5, 0.0035;<br><br>IL-5 d5, <0.0001;<br>IL-5 d6, <0.0001	Methods
Fig S6g	Student's T-test	Methods	(N) 5<br>(5) 5	T cells from the lungs of individual mice stimulated with PMA and ionomycin	Methods	Individual mice with mean	Figure legend	IL-13 d5 *vs.* N, 0.2982	Methods
Fig S6h	Student's T-test	Methods	(N) 5<br>(5) 5	NK cells from the lungs of individual mice stimulated with PMA and ionomycin	Methods	Individual mice with mean	Figure legend	IL-13 d5 *vs.* N, 0.4127	Methods
Fig S6j	One-Way ANOVA, Bonferroni post-test	Methods	(N) 6<br>(1) 6<br>(2) 6<br>(9) 5	Treg cells from the lungs of individual mice	Methods	Individual mice with mean	Figure legend	Treg frequency N *vs.* d1, >0.9999;<br>Treg frequency N *vs.* d2, 0.5481;<br>Treg frequency N *vs.* d9. 0.9347	Methods
Fig S7d	One-Way ANOVA, Bonferroni post-test	Methods	6 per group	BAL supernatants from individual *Rag2*$^{-/-}$ *Il2rγc*$^{-/-}$, ILC2 transferred *Rag2*$^{-/-}$ *Il2rγc*$^{-/-}$ and C57BL6 mice	Figure Legend	Error bars represent Mean ± SEM	Figure Legend	CCL17 *Rag2*$^{-/-}$ *Il2rγc*$^{-/-}$ *vs* WT d2, 0.0001;<br>CCL17 ILC2 transferred *Rag2*$^{-/-}$ *Il2rγc*$^{-/-}$ *vs* *Rag2*$^{-/-}$ *Il2rγc*$^{-/-}$ *vs* d2, 0.0289;<br>CCL17 *Rag2*$^{-/-}$ *Il2rγc*$^{-/-}$ *vs* WT d6, 0.0329;<br>CCL17 ILC2 transferred *Rag2*$^{-/-}$ *Il2rγc*$^{-/-}$ *vs* *Rag2*$^{-/-}$ *Il2rγc*$^{-/-}$ *vs* d6, 0.0468;<br><br>BRP39 *Rag2*$^{-/-}$ *Il2rγc*$^{-/-}$ *vs* WT d6, 0.0232;<br>BRP39 ILC2 transferred *Rag2*$^{-/-}$ *Il2rγc*$^{-/-}$ *vs* *Rag2*$^{-/-}$ *Il2rγc*$^{-/-}$ *vs* d6, 0.0234;	Methods
Fig S7e	Two-Way ANOVA, Bonferroni post-test	Methods	3 per group	BMDM cells from individual *Rag2*$^{-/-}$ *Il2rγc*$^{-/-}$*vs* WT C57BL6 mice	Figure Legend	Error bars represent Mean ± SEM	Figure Legend	IL13 (0) + IL33 (0) *vs.* IL13 (0) + IL33 (2.5), 0.0034;<br>IL13 (0) + IL33 (0) *vs.* IL13 (0) + IL33 (10), 0.0360;<br>IL13 (2.5) + IL33 (0) *vs.* IL13 (2.5) + IL33 (10), 0.0448;<br>IL13 (10) + IL33 (0) *vs.* IL13 (10) + IL33 (10), 0.0208	
Fig S7g	One-Way ANOVA, Bonferroni post-test	Methods	4 per group	BMDM cells from individual *Rag2*$^{-/-}$ *Il2rγc*$^{-/-}$*vs* WT C57BL6 mice	Figure Legend	Error bars represent Mean ± SEM	Figure Legend	CCL17 IL-13(0) + IL-33(0) *vs.* IL-13(10) + IL-33(10), 0.0016;<br>IGF-1 IL-13(0) + IL-33(0) *vs.* IL-13(10) + IL-33(10), 0.0249;<br>HGF IL-13(0) + IL-33(0) *vs.* IL-13(10) + IL-33(10), 0.0053;	Methods
