# Supplementary material for: IL-33-ST2 axis regulates myeloid cell differentiation and activation enabling effective club cell regeneration
Source: Nat Commun. 2020 Sep 22;11:4786. doi: 10.1038/s41467-020-18466-w (PMC7508874; doi:10.1038/s41467-020-18466-w)
Supplement: Supplementary file 3 — Reporting Summary [file 41467_2020_18466_MOESM3_ESM.pdf]

nature research

NCOMMS-19-15482E
Corresponding author(s): Alison A Humbles
Marina Pretolani

Reporting Summary

Nature Research wishes to improve the reproducibility of the work that we publish. This form provides structure for consistency and transparency in reporting. For further information on Nature Research policies, see Authors & Referees and the Editorial Policy Checklist.

Statistical parameters

When statistical analyses are reported, confirm that the following items are present in the relevant location (e.g. figure legend, table legend, main text, or Methods section).

n/a	Confirmed	
☐	☒	The exact sample size (*n*) for each experimental group/condition, given as a discrete number and unit of measurement
☐	☒	An indication of whether measurements were taken from distinct samples or whether the same sample was measured repeatedly
☐	☒	The statistical test(s) used AND whether they are one- or two-sided<br>*Only common tests should be described solely by name; describe more complex techniques in the Methods section.*
☐	☒	A description of all covariates tested
☐	☒	A description of any assumptions or corrections, such as tests of normality and adjustment for multiple comparisons
☐	☒	A full description of the statistics including central tendency (e.g. means) or other basic estimates (e.g. regression coefficient) AND variation (e.g. standard deviation) or associated estimates of uncertainty (e.g. confidence intervals)
☐	☒	For null hypothesis testing, the test statistic (e.g. *F*, *t*, *r*) with confidence intervals, effect sizes, degrees of freedom and *P* value noted<br>*Give P values as exact values whenever suitable.*
☒	☐	For Bayesian analysis, information on the choice of priors and Markov chain Monte Carlo settings
☐	☒	For hierarchical and complex designs, identification of the appropriate level for tests and full reporting of outcomes
☒	☐	Estimates of effect sizes (e.g. Cohen's *d*, Pearson's *r*), indicating how they were calculated
☐	☒	Clearly defined error bars<br>*State explicitly what error bars represent (e.g. SD, SE, CI)*

Our web collection on statistics for biologists may be useful.

Software and code

Policy information about availability of computer code

Data collection	FastQC; Hisat2 (version 2.0.2); Cell Ranger version 2.0 pipeline (10x Genomics);
Data analysis	Seurat package V2.2 and R 3.4; UCSC Cell Browser; metabase R package (v4.2.0, Clarivate Analytics); GenePattern; GraphPad Prism v8.0.1.

For manuscripts utilizing custom algorithms or software that are central to the research but not yet described in published literature, software must be made available to editors/reviewers upon request. We strongly encourage code deposition in a community repository (e.g. GitHub). See the Nature Research guidelines for submitting code & software for further information.

Data

Policy information about availability of data

All manuscripts must include a data availability statement. This statement should provide the following information, where applicable:

- Accession codes, unique identifiers, or web links for publicly available datasets
- A list of figures that have associated raw data
- A description of any restrictions on data availability

We have uploaded the RNA sequencing data (in figures 3, 4 and 6) to Gene Expression Omnibus (GEO). The data has been deposited in the Gene Expression Omnibus with accession numbers GSE155356, GSE155359 and GSE155261.

Field-specific reporting

Please select the best fit for your research. If you are not sure, read the appropriate sections before making your selection.

☒ Life sciences ☐ Behavioural & social sciences ☐ Ecological, evolutionary & environmental sciences

For a reference copy of the document with all sections, see nature.com/authors/policies/ReportingSummary-flat.pdf

Life sciences study design

All studies must disclose on these points even when the disclosure is negative.

Sample size	Sample size for each individual experiment was usually 3-8 mice per group. All experiments reported have n number and repetitions reported in the figure legend.<br><br>Initially, we run pilot studies to characterize knockout mice and antibodies that are necessary to establish a treatment effect and to prove efficacy in the comparison of KO and/or antibody treatment groups to wild type and non-treated groups of mice. Our pilot experiment confirmed that groups with 5-8 mice per group x 2 repeats for variability, are sufficient to ensure reproducibility in our study. No statistical tests were used to determine sample size.
Data exclusions	No data exclusion criteria were laid out and have thus not been included in the manuscript.
Replication	Experiments were then repeated 2-3 times independently and results were pooled. Some difficulty occurred in experiments requiring genetic knock-outs as often group size was determined by the availability of mice. Experiments requiring the pooling of samples also ran into sample number limitations.<br>All attempts at replication were successful, and standard deviations were within expected ranges.
Randomization	Mice were randomized based on weight prior to experiment set up.
Blinding	Where feasible, test samples were blinded to the operator, i.e. mRNA and protein analyses. Immunohistochemical scoring was also performed blinded. In some cases, blinding of test samples was not applicable, as samples were processed identically through standard and in some cases automated procedures (RNA sequencing and analyses) but these processes do not facilitate bias outcomes.

Reporting for specific materials, systems and methods

Materials & experimental systems

n/a	Involved in the study	
☒	☐	Unique biological materials
☐	☒	Antibodies
☒	☐	Eukaryotic cell lines
☒	☐	Palaeontology
☐	☒	Animals and other organisms
☒	☐	Human research participants

Methods

n/a	Involved in the study	
☒	☐	ChIP-seq
☐	☒	Flow cytometry
☒	☐	MRI-based neuroimaging

Antibodies

Antibodies used

All antibodies used in this study are detailed in either the methods (transfer of lung ILC2s to Rag2-/-/Il2rg-/- mice, identification of ILCs, Ex vivo stimulation of ILCs) or in Supplementary Tables 1 and 2 of the manuscript.

F4/80, Rat IgG2b, CL:A3-1, 1:200, #ab6640 ,abcam
CCSP, Rabbit Serum, 1:5000, #WRAB-3950, Seven Hills
Ki67-APC, Rat IgG2a, SolA15, 1:200, 1#7-5698-82, eBioscience
IL-33, Goat IgG Polyclonal, 1:200, #AF3626, R&D systems
ß-tubulin-Fitc, Mouse IgG1, TUB 2.1, 1:200, #ab11310 abcam
CD3ε-biotin, Hamster IgG, 145-2C11, 25μg/mL, #100304 Biolegend
CD19-biotin, Rat IgG2a, 1D3, 25μg/mL, #553784 BD biosciences
B220-biotin, Rat IgG2a, RA3-6B2, 25μg/mL, #103204 Biolegend
CD5-biotin, Rat IgG2a, 53-7.3, 25μg/mL, #100604 Biolegend
TCRß-biotin, Hamster IgG, H57-597, 25μg/mL, #109204 Biolegend
TCRγδ-biotin, Hamster IgG, GL3, 25μg/mL, #118103 Biolegend
CD11c-biotin, Hamster IgG, N418, 25μg/mL, #117304 Biolegend
F4/80-biotin, Rat IgG2a, BM8, 25μg/mL, #123106 Biolegend
Gr-1-biotin, Rat IgG2b, RB6-8C5, 25μg/mL , #108404 Biolegend
Ter119-biotin, Rat IgG2b, TER-119, 25μg/mL, #116204 Biolegend
CD49b-biotin, Rat IgM, DX5, 25μg/mL, #108904 Biolegend
NK1.1-biotin, Mouse IgG2a, PK136, 25μg/mL, #108704 Biolegend
CD27-biotin, Hamster IgG, LG3A10, 25μg/mL, #124206 Biolegend
Secondary anti-Goat IgG-Alexa Fluor 488, Donkey IgG, Polyclonal, 2μg/mL, #A32814 ThermoFisher
Secondary anti-Goat IgG-Alexa Fluor 555, Donkey IgG, Polyclonal, 2μg/mL, #A32816 ThermoFisher
Secondary anti-Rabbit IgG-Alexa Fluor 488 ,Donkey IgG, Polyclonal, 2μg/mL, #A32790 ThermoFisher
Secondary anti-Rabbit IgG-Alexa Fluor 555, Donkey IgG, Polyclonal, 2μg/mL, #A32794 ThermoFisher

CD45-BUV395, Rat IgG2b, 30-F11, 1:200, #564279 BD Bioscience
F4/80-APC Cy7, Rat IgG2a, BM8, 1:50, #123118 Biolegend
CD11c-APC, Hamster IgG1, HL3, 1:200, #550261 BD Bioscience
CD11b-BV421, Rat IgG2b, M1/70, 1:200, #101251 Biolegend
CD11b-PE, Rat IgG2b, M1/70, 1:200 #101208, Biolegend
Ly6C-PE CY7, Rat IgM, AL-21, 1:200 #560593, BD Bioscience
CD64-Alexa Fluor 594, Rat IgG2a, 290322, 5μL/106 cells, #FAB20741P R&D systems
Ki67-APC, Rat IgG2a, SolA15, 1:200, #17-5698-82 eBioscience
Arg-1-PE, Sheep IgG, Polyclonal, 5μL/106 cells, #IC5868P R&D systems
Biotinylated CD206, Goat IgG, Polyclonal, 5μg/mL, #BAF2535 R&D systems
CD206-BV650, Rat IgG2a, C068C2, 1:200, #141723 Biolegend
Biotinylated YM1, Goat IgG, Polyclonal, 1:200, #BAF2446 R&D systems
Biotinylated FIZZ-1, Goat IgG, Polyclonal, 1:200, #BAF1523 R&D systems
Siglec-F-BV711, Rat IgG2a, E50-2440, 1:200, #740764 BD Bioscience
ST2-Fitc, Rat IgG1, DJ8, 1:200, #101001F MD Biosciences
Streptavidin-BV421, - - 1:1000, #405226 Biolegend
CD3ε-PE, Hamster IgG1, 145-2C11, 1:100, #553063 BD bioscience
CD3ε- BUV395, Hamster IgG1, 145-2C11, 1:100, #563565 BD bioscience
CD49b-PE/Dazzle 594, Rat IgM, DX5, 1:200, #108924 Biolegend
CD45-Alexa Fluor 700, Rat IgG2b, 30-F11, 1:400, #103128 Biolegend
CD45-BV510, Rat IgG2b, 30-F11, 1:300, #103137 Biolegend
CD25-BV650, Rat IgG1, PC61, 1:400, #102037 Biolegend
CD90-APC eFluor 780, Rat IgG2a, 53-2.1, 1:400, #47-0902 eBioscience
CD44-Alexa 700, Rat IgG2b, IM7, 1:400, #103026 Biolegend
CD4-BV750, Rat IgG2b, GK1.5, 1:200, #100467 Biolegend
CD8-PE Cy5, Rat IgG2a, 56-6.7, 1:200, #100710 Biolegend
CD62L-BV421, Rat IgG2a, MEL-14, 1:200, #104435 Biolegend
IL-18Rα-PerCP eFluor 710, Rat IgG2a, P3TUNYA, 1:100, #46-5183 eBioscience
CD90-eFluor 450/BV421, Mouse IgG2a, 53-2.1, 1:200, #48-0900-82 eBioscience
CD45-eFluor 450/BV421, Rat IgG2b, 30-F11, 1:200, #48-0451-82 eBioscience
CD3ε-eFluor 450/BV421, Hamster IgG1, 145-2C11, 1:200, #562600 BD Bioscience
TCRß-eFluor 450/BV421, Hamster IgG1, H57-597, 1:200, #48-5961-82 eBioscience
TCRγδ-eFluor 450/BV421, Hamster IgG1, GL3, 1:200, #118120 Biolegend
CD5-eFluor 450/BV42,1 Rat IgG2a, 53-7.3, 1:200, #100617 Biolegend
F4/80-eFluor 450/BV421, Rat IgG2a, BM8, 1:200, #123131 Biolegend

	CD11c-eFluor 450/BV421, Hamster IgG, N418, 1:200, #117329 Biolegend<br>Gr-1-eFluor 450/BV421, Rat IgG2b, RB6-8C5, 1:200, #108433 Biolegend<br>CD19-eFluor 450/BV421, Rat IgG2a, 1D3, 1:200, #562701 BD Bioscience<br>FCRI-eFluor 450/BV421, Hamster IgG, MAR-1, 1:200, #48-5898-82 eBioscience<br>B220-eFluor 450/BV421, Rat IgG2a, RA3-6B2, 1:200, #103239 Biolegend<br>NK1.1-eFluor 450/BV421, Mouse IgG2a, PK136, 1:200, #108731 Biolegend<br>CD27-eFluor 450/BV421, Hamster IgG, LG3A10, 1:200, #561245 BD Bioscience<br>FoxP3-Alexa Fluor 647, Rat IgG2b, MF23, 1:200, #560401 BD Bioscience<br>GATA-3-eFluor 660, Rat IgG2b, TWAJ, 1:400, #50-9966-42 eBioscience<br>IL-13-PE, Rat IgG1 clone 13A, 1:200, #12-7133-41 eBioscience<br>IL-13- eFluor 660, Rat IgG1, clone 13A, 1:200, #50-7133-82 eBioscience
Validation	All used antibodies were validated by supplier and reported in recent publications:<br>Nat Immunol. 2016 Jun;17(6):646-55<br>Nat Immunol. 2016 Jun;17(6):626-35<br>Immunity. 2015 Mar 17;42(3):566-7<br>Cell Rep. 2017 Apr 11;19(2):246-254<br>Stem Cells. 2009 Mar;27(3):612-22

Animals and other organisms

Policy information about studies involving animals; ARRIVE guidelines recommended for reporting animal research

Laboratory animals	For animals used in France, animals were housed under pathogen-free conditions, according to protocols approved by the Paris Nord Ethic Committee for Animal Care and Ministry of Education and Reserach (no.02013.01). For animals in the US, they were housed at MedImmune/AstraZeneca and treated according to protocols approved by the Institutional Animal Care and Use Committee at MedImmune.<br><br>C57BL/6J, Rag2-/-/Il2rgc-/- (Rag2tm1Fwa Il2rgtm1Wjl) deficient (Taconic, #4111), green fluorescent protein (GFP) transgenic (C57BL/6-Tg(CAG-EGFP)131Osb/LeySopJ, Jax, #006567) mice, and ST2-GFP (C57BL/6-Il1rl1tm3548.1(T2a-EGFP)Arte/+) mice, were randomized and placed into groups based on weight. Female (age 8–13 weeks at start of experiment) mice were used and the control mice were sex matched with the group of interest. All mice were housed in a specific pathogen-free environment and kept in a room with controlled temperature (~23 °C) and humidity under 12 h light/dark cycle.<br><br>After naphthalene injection, animals were monitored daily for signs of distress and were given gel packs to aid in the recovery.
Wild animals	No wild animals were used in the study.
Field-collected samples	No field collected samples were used in the study.

Flow Cytometry

Plots

Confirm that:

- [x] The axis labels state the marker and fluorochrome used (e.g. CD4-FITC).
- [x] The axis scales are clearly visible. Include numbers along axes only for bottom left plot of group (a 'group' is an analysis of identical markers).
- [x] All plots are contour plots with outliers or pseudocolor plots.
- [x] A numerical value for number of cells or percentage (with statistics) is provided.

Methodology

Sample preparation	Lung cell suspension:<br>Lungs were perfused with PBS, cut into ~2mm pieces and incubated with LiberaseTM (42.4 µg/mL) and DNAseI (10U/mL; both from Roche) for 45-60 minutes at 37oC before being mashed through a 70 µm cell strainer and washed with complete RPMI. Remaining blood cells were lysed with ACK cell lysing buffer (Invitrogen) and single cell suspensions were obtained. After centrifugation, cells were counted and resupended in FACS staining buffer at 1 million per mL.<br><br>BAL cell preparation:<br>Mice were terminally anesthesized by a subcutaneous injection of ketamine and xylazine, as described above. BAL fluid was obtained by injecting and recovering 5 x 1 mL of sterile PBS through a tracheal catheter. After centrifugation (400 g, 5 min, 4°C), cell pellets were counted and resuspended in FACS staining buffer at 0.5 million per mL.

Instrument	FACS data were acquired using BD LSRFortessa™ Flow Cytometer
Software	Flow Jo 9.5 and BD FACS DIVA softwares were used for analysis
Cell population abundance	The abundance of cells was dependent upon cell type and sorting condition.<br>Resident AAM macrophages were typically over 40% of BAL cells on d9 after naphthalene injury.<br>After depletion AAM cells numbers dropped by 8 fold on d9 after naphthalene injury.<br>ST2-/- and Rag2-/-/Il2rgc-/- AAM cells counts also dropped by 3 fold on d9 after naphthalene injury.<br>ILC2 cells were typically less then 1% in naphthalene-treated mice (day 3 post-treatment).<br>Bone marrow cells from C57BL/6J, Balb/c, ST2-GFP, or ST2-/- mice on the Balb/c background, were seeded at 400 000 cells per well in 24-well plate. After 6 days, over 50% of bone marrow-derived macrophages (BMM) displayed AAM phenotype in C57BL/6J, Balb/c, ST2-GFP, whereas BMDMs from ST2-/- mice (on the Balb/c background) exhibited 10% AAM phenotype.
Gating strategy	Negative control (unstained) and fluorophore-positive cells were used to establish gates for each cell type. Gates were drawn to collect cells expressing either fluorophore.<br>Gating strategy for myeloid, ILCs, T, NK and Treg cell characterization are presented in supplementary Figures 1 and 6

☒ Tick this box to confirm that a figure exemplifying the gating strategy is provided in the Supplementary Information.
