# Supplementary material for: IL-33-ST2 axis regulates myeloid cell differentiation and activation enabling effective club cell regeneration
Source: Nat Commun. 2020 Sep 22;11:4786. doi: 10.1038/s41467-020-18466-w (PMC7508874; doi:10.1038/s41467-020-18466-w)
Supplement: Supplementary file 4 — Description of Additional Supplementary Files [file 41467_2020_18466_MOESM4_ESM.pdf]

Description of Additional Supplementary Files

Supplementary Data 1
Group comparison result for WT, P2 vs P1

Supplementary Data 2
Group comparison result for WT, P3 vs P1

Supplementary Data 3
Group comparison result for WT, P3 vs P2

Supplementary Data 4
Group comparison result for WT, P3 d6 vs d0

Supplementary Data 5
Representative differentially expressed genes between P1, P2 and P3 in each cluster as heatmap of Figure 3b

Supplementary Data 6
Summary for Geneset enrichment analysis (Figure 3b, 3d)

Supplementary Data 7
Group comparison result (P2 vs P3) for the complete list for Growth Factors, ECM and inflammation (Figure 3c)

Supplementary Data 8
Group comparison result for P1, ST2KO vs WT

Supplementary Data 9
Group comparison result for P2, ST2KO vs WT

Supplementary Data 10
Group comparison result for P3, ST2KO vs WT

Supplementary Data 11
Summary for pathway enrichment analysis P2, ST2KO vs WT (Figure 6b)

Supplementary Data 12
Summary for pathway enrichment analysis P3, ST2KO vs WT (Figure 6c)

Supplementary Data 13
Group comparison result for the complete list for Growth Factors, ECM and immune response, P2 ST2KO vs WT (Figure 6d)

Supplementary Data 14
Group comparison result for the complete list for Growth Factors, ECM and immune response, P3 ST2KO vs WT (Figure 6d)
